# Determination of genomic regions associated with early storage root formation and bulking in cassava

**DOI:** 10.3389/fpls.2024.1391452

**Published:** 2024-06-26

**Authors:** Simon Peter Abah, Joseph Okpani Mbe, Daniel Kwadjo Dzidzienyo, Damian Njoku, Joseph Onyeka, Eric Yirenkyi Danquah, Samuel Kwane Offei, Peter Kulakow, Chiedozie Ngozi Egesi

**Affiliations:** ^1^ Bioscience, National Root Crops Research Institute, Umudike, Nigeria; ^2^ Cassava Breeding, International Institute for Tropical Agriculture, Ibadan, Nigeria; ^3^ West African Centers for Crop Improvement, University of Ghana, Accra, Ghana; ^4^ Biotechnology Centre, University of Ghana, Accra, Ghana

**Keywords:** cassava, early bulking and formation, genomic region, storage root, SNP markers, productivity

## Abstract

Early cassava storage root formation and bulking is a medium of escape that farmers and processors tend to adopt in cases of abiotic and biotic stresses like drought, flood, and destruction by domestic animals. In this study, 220 cassava genotypes from the International Institute of Tropical Agriculture (IITA), National Root Crops Research Institute (NRCRI), International Center for Tropical Agriculture (CIAT), local farmers (from farmer’s field), and NextGen project were evaluated in three locations (Umudike, Benue, and Ikenne). The trials were laid out using a split plot in a randomized incomplete block design (alpha lattice) with two replications in 2 years. The storage roots for each plant genotype were sampled or harvested at 3, 6, 9, and 12 month after planting (MAP). All data collected were analyzed using the R-statistical package. The result showed moderate to high heritability among the traits, and there were significant differences (*p*< 0.05) among the performances of the genotypes. The genome-wide association mapping using the BLINK model detected 45 single-nucleotide polymorphism (SNP) markers significantly associated with the four early storage root bulking and formation traits on Chromosomes 1, 2, 3, 4, 5, 6, 8, 9, 10, 13, 14, 17, and 18. A total of 199 putative candidate genes were found to be directly linked to early storage root bulking and formation. The functions of these candidate genes were further characterized to regulate i) phytohormone biosynthesis, ii) cellular growth and development, and iii) biosynthesis of secondary metabolites for accumulation of starch and defense. Genome-wide association study (GWAS) also revealed the presence of four pleiotropic SNPs, which control starch content, dry matter content, dry yield, and bulking and formation index. The information on the GWAS could be used to develop improved cassava cultivars by breeders. Five genotypes (W940006, NR090146, TMS982123, TMS13F1060P0014, and NR010161) were selected as the best early storage root bulking and formation genotypes across the plant age. These selected cultivars should be used as sources of early storage root bulking and formation in future breeding programs.

## 1 Introduction

Cassava (*Manihot esculenta* Crantz) is a shrub and one of the most crucial food plants growing in tropical regions (Montagnac et al., 2009). It is an extensive source of calories for more than 600 million human beings worldwide. After rice, wheat, and maize, it ranks as the fourth most important staple crop on the planet (FAO, 2007). An estimated 60% of Africans rely upon the crop as a widespread supply of calories (FAOSTAT, 2019). In Africa, in terms of production, it ranks first followed by maize, plantain, and rice; in Nigeria, cassava is seen as the third most important staple food with high calories after rice and maize (Nweke et al., 2002; FAOSTAT, 2019). Nigeria is the largest producer of cassava in Africa (FAO, 2014; FAOSTAT, 2019), and over 90% of Nigeria’s farming populace depend on the crop (Nweke et al., 2002).

Despite the significance of cassava, there are still difficult problems that affect the growth and production performance of the crops and lead to a decline in the adoption rate and, most of the time, rejection. According to Ekanayake et al. (1998) and Osei (2005), the environment and growing conditions have an impact on the cassava cycle. Wahid et al. (2007) demonstrated that phenological stages and genotypes both influence how a plant responds to environmental stress situations. El-Sharkawy (2004) and Alves (2002a, b) claimed that the partitioning of features, such as dry matter, that are orientated toward either root or shoot production is influenced by the environment. As a result, different cassava genotypes mature at different periods, with some maturing earlier than others (Okogbenin and Fregene, 2002; Okogbenin et al., 2008). For instance, cassava can be grown for up to 2 years in cooler or drier settings but can be harvested as early as 6 months in hot, humid conditions. These hot, arid climates are frequently characterized by a short dry season of up to 7 months, followed by a rainy season lasting 4 to 5 months. Therefore, farmers preferred adopting cultivars that are ready during the short growing season. According to Annor-Frempong (1994), “early maturing” was the most frequently mentioned quality that farmers in Ghana’s ecological transition zone requested. According to several studies (Annor-Frempong, 1994; Kamau et al., 2011), late bulking is a significant factor in the low adoption and rejection of cassava cultivars in African nations. Late-bulking cultivars cannot be successfully used for the sequential cultivation of other crops since they occupy farmers’ land for lengthy periods of time and are also very expensive to maintain for lengthy periods in the field.

According to Okogbeni et al. (2013a, b), early storage root yield (bulking) in cassava is also shown to be a crucial characteristic of drought tolerance. Early planting is a key control strategy for the cassava brown streak disease (CBSD), which is presently widespread in eastern, central, and southern Africa (Kamau et al., 2011; Legg et al., 2011). Studies have shown that early planting helps cassava varieties escape drought due to late season, infestation from pests, and occurrence of diseases (Osei, 2005; Adjebeng-Danquah et al., 2012). Early bulking is also very important in situations where farmers, especially in semi-arid areas, are forced to boost production and harvest their crops after just one cycle of rain due to pressure on agricultural fields. However, a report by Adjebeng-Danquah et al. (2012) indicated that excessive earliness affects yield due to the reduction in the time of dry matter accumulation. To reduce yield loss due to early harvesting, it is important to carefully select cassava genotypes that partition dry matter into the storage roots earlier.

The main focus of population geneticists and conservation biologists is to better understand the size and distribution of genetic diversity and also the evolutionary processes that have shaped this diversity (Hartl and Clark, 1997). Genetic diversity and composition of populations are influenced by a number of evolutionary processes, including recombination, mutation, migration, genetic drift, and natural selection.

Diseases and the introduction of new cassava varieties to Africa have an impact on population distribution and reduce genetic diversity, whereas gene flow between cassava populations that happens through seeds, pollen, or actual movement of plants between localities managed by farmers may increase genetic diversity in populations (Tewodros and Zelalem, 2015).

According to Hamrick and Godt (1997), molecular markers offer a way to precisely quantify the genetic diversity and genetic structure of a population. They are also acknowledged as important tools to guide the management of plant genetic resource conservation.

For a very long time, scientists have investigated molecular diversity in plants as a useful component of genetic studies. According to Wu and Tanksley (1993), the best method for determining genetic diversity and differentiation as well as the relative strength of the many forces influencing the variety is variation in allele frequency at numerous unrelated loci. Numerous studies have also demonstrated how DNA markers can be used to identify genetic variability in cassava. Restriction fragment length polymorphisms (RFLPs) (Beeching et al., 1993), random amplified polymorphic DNA (RAPD) markers (Marmey et al., 1994), and simple sequence repeat (SSR) markers (Fregene et al., 2003; I, II) have all been used to assess the genetic diversity of African cassava. This may understate the genetic diversity observed in farmer fields and the likelihood that there are numerous recent improvements that are well-known in research institutes. Nevertheless, the majority of cassava diversity studies have outlined how the crop differs from its Latin American origins and genetically from other regions where it has been introduced, such as Africa. When choosing locations for plant exploration and germplasm collection for breeding programs, it helps to be aware of the geographic patterns of diversity for the crop. The characterization of farmer-specific cultivars at the molecular level aids researchers in better understanding and focusing any upcoming assistance on the needs and preferences of the farmers.

Farmers often choose vigorous seedlings from spontabeneous germination; by doing so, they may unintentionally be choosing a localized heterozygous genotype. A few farmers were also involved in cassava planting material distribution and mitigation programs run by government and non-governmental organizations. Thus, it is normal to discover a number of local varieties that have been farmed continuously for more than one farmer generation in the area, as well as local and improved types that may be sweet, bitter, or both, in a typical small-scale farmer’s field (Berthaud et al., 2001).

Similar to those in Ghana (Manu-Aduening et al., 2005), we discovered many local varieties in Nigeria in the fields of small-scale farmers. These varieties are genetically fairly heterogeneous and the product of dynamic evolution involving both natural and human selection (Jarvis and Hodgkin, 1999).

Since selection could now be conducted early in the growing cycle, which can even be conducted at the seedling stage, the development of molecular DNA markers has allowed genome-wide studies and plant genetic transformation, which offer promising solutions to the breeding challenges of lonmarkers such as isozymesg growth cycles. The use of DNA markers is based on the identification of naturally occurring DNA sequence polymorphisms in various individuals of a species. DNA markers employ indirect selection to identify suitable genotypes for desirable quantitative traits like dry matter content (DMC) before such variables can be evaluated phenotypically, in contrast to formal breeding approaches that only rely on direct selection by phenotypic effect. All tissues have DNA markers, which can be detected and are not influenced by the environment.

Molecular methods are now at the forefront and useful tools for the majority of biological research, including fundamental, adaptive, and applied research. Understanding genetic markers as an essential breeding tool has given us information about the extent of natural variation and how it is passed along. Early cassava markers included morphological markers (Graner, 1942; Hershey and Ocampo, 1989), which were then followed by biochemical markers such as isozymes (Hussain and Hayakawa, 1987; Campo et al., 1992; Lefevre and Charrier, 1993a, 1993b). These markers are constrained by the environment and the interaction between genotype and environment, which may make it difficult to accurately detect duplicates in genotypes (Collard et al., 2005). Therefore, molecular markers may be more reliable in genetic diversity studies for characterizing accessions strongly related to morphological characteristics (Collard et al., 2005). Different molecular markers such as RFLPs, RAPDs, amplified fragment length polymorphism (AFLP), SSRs, and single-nucleotide polymorphism (SNP) have all been used to assess genetic diversity in cassava germplasm (Beeching et al., 1993; Fregene et al., 2000; Kawuki et al., 2009; Asare et al., 2011; Okogbenin et al., 2012; da Silva et al., 2015). Despite the fact that SSR (microsatellites) and SNP markers are the most competitive ones for diversity studies, SNP markers are easier to assay per locus and are the most common marker system in plant, animal, and microorganism genomes in comparison to SSR, which has limited stutter bands, making scoring difficult (Park et al., 2009). SNP markers are regarded as the new generation molecular markers for diverse applications in identifying and differentiating specific genetic variations even in a low diversity species because of their adaptability (Ferri et al., 2010).

Recent research by Okogbenin et al. (2012) and da Silva et al. (2015) demonstrated that SNP markers have a much higher and faster accuracy in the study of genetic diversity and gains in selection than the conventional approaches alone. When compared to other important crops like maize and rice, the use of molecular markers in the study of cassava variety and gain is a quick and innovative technique that has not yet been completely utilized. More research that could aid in the genetic enhancement of cassava is necessary as a result of the economic significance of the crop for food security, particularly in Africa. Accordingly, the objectives of this study were to i) assess the genetic diversity of cassava roots in Nigeria, ii) assess the genetic composition and growing condition of cassava storage root roots in different agroecological zones, and iii) identify the genomic regions and SNPs linked to natural variations for early storage root formation and bulking (ESRFB) traits in cassava.

## 2 Materials and methods

### 2.1 Study area and planting materials

**Figure 1:**
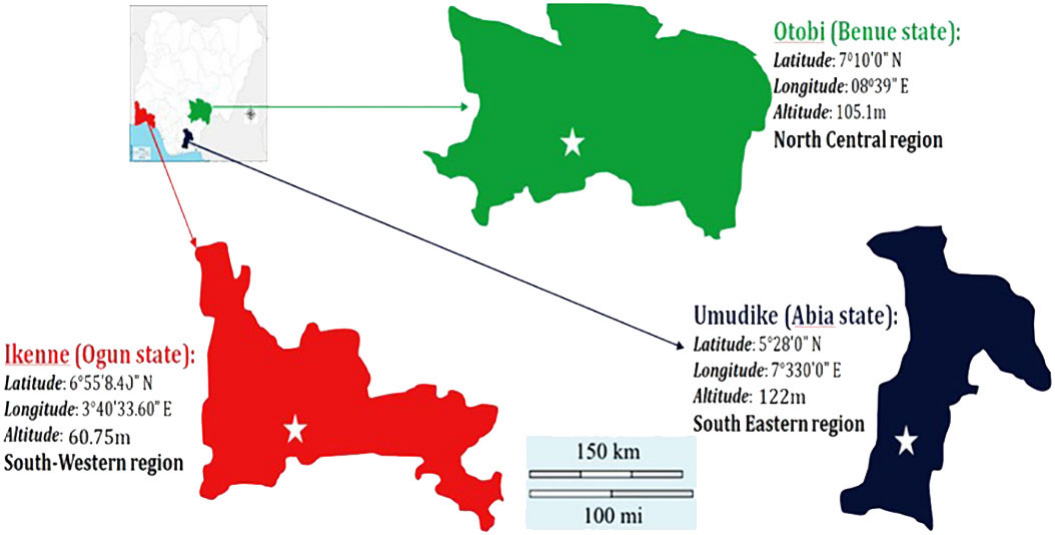
The map of the study areas.

A diverse training population of 220 cassava genotypes, which comprised of materials from the International Institute of Tropical Agriculture (IITA), National Root Crops Research Institute (NRCRI), International Center for Tropical Agriculture (CIAT), local farmers (from farmer’s field), and NextGen Cassava Breeding Project, was used. These planting materials were evaluated in three locations (Umudike, Benue, and Ikenne). Umudike is located in the southeastern region with latitude of 5°28′0″N, longitude of 7°330′0″E, and altitude of 122 m above sea level. Benue is located in the north central region with latitude of 7°47′0″N and longitude of 10°0′0″E. Ikenne is located in the southwestern region with latitude of 6°55′8.40″N, longitude of 3°40′33.60″E, and altitude of 59 m above sea level in Nigeria. The map is shown in **Figure 1**.

### 2.2 Field layout and experimental design

**Figure 2:**
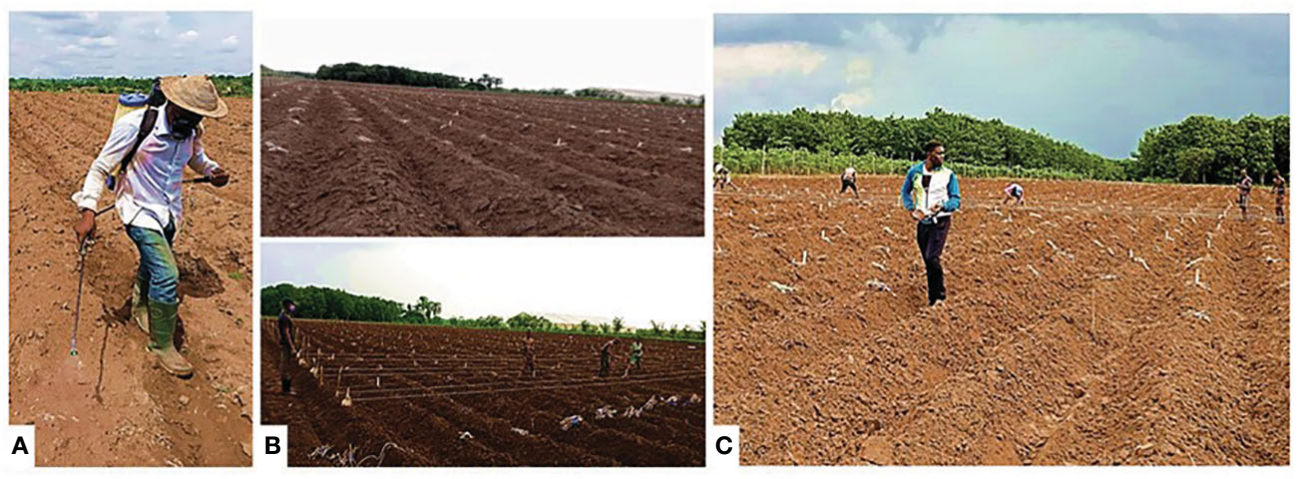
**(A)** Land preparations. **(B)** Marking. **(C)** Laying of planting materials.

The trials were laid out using a split plot in a randomized incomplete block design (alpha lattice) with two replications at three locations in 2 years. The main plot was the set of time at harvest (3, 6, 9, and 12 MAP), while the subplot was the 220 genotypes. The plot size was 0.8 × 5 m with 20-cm cuttings of genotypes planted on ridges at an interspacing of 1 m by intraspacing of 0.8 m. The trials were established between 2019/2020 and 2021/2022 cropping seasons as shown in **Figure 2**.

### 2.3 Phenotypic measurements

In order to identify the patterns of ESRFB in cassava genotypes, storage roots from each plant genotype were sampled or harvested at 3, 6, 9, and 12 MAP. At each sampling point under the destructive phenotyping, data were collected from three plant stands using below-ground parts. The following cassava storage root traits were measured across the four sets of harvesting times (3, 6, 9, and 12 MAP): storage root system length, breadth, and depth; total root weight; root architecture; root size (root length and breadth); root density; number of root; root shape; root surface texture; external root back appearance; and pedunculation. Plant phenotyping was conducted at the stage of tuberization (3 MAP) to the stage of nutrient development (6 MAP), and also to the stage of early maturity (9 MAP) to full maturity (12 MAP) according to the set of splits.

Other derivative traits that were calculated using the measured storage traits include fresh root yield (FRYD), harvest index (HI), dry matter content (DMC), starch content (SC), root density (rt_density), and root size.

#### 2.3.1 Measurement of storage root system and architectural traits

The formula used was adopted from the wiki ontology developed by the NextGen breeding project cassava (https://www.cassavabase.org/wiki/ontology).

Root weight (kg): This measurement is taken using a balance scale in unit of kilogram.

Root architecture: This is the measurement of the multiple root orientations. Orientation can be classified using the following: parallel to the surface (0°), perpendicular to the surface (90°), and diagonal to the surface (45°).

Root size characteristics: This is a measurement of total root length and three root diameters taken at equal intervals across each root to account for root tapering, which is as follows: *short*, root less than 40 cm; *normal*, root between 40 cm and 80 cm; and *long*, root greater than 80 cm.

Number of roots: This is conducted by counting the number of basal roots to storage roots, and the number of nodal roots to storage roots.

Root shape: This is a categorical trait that can be classified as conical, cylindro-conical, cylindrical, and globular (spherical shape).

Root surface texture: This trait can be classified as smooth, medium, or rough surface texture.

External bark appearance: This can be classified as gray thin back, gray thick back, brown thin back, and brown thick back.

Pedunculation: The presence of neck/peduncle in the tuber leads to longer shelf life. This can be classified as present or absent root peduncle.

#### 2.3.2 Calculation of the derivative traits

The traits used in this study were adopted from the trait ontology of breedbase.org and cassavabase.org (https://www.cassavabase.org/search/quick?term=ontology). (Equations 1–10)

##### 2.3.2.1 DMC

In order to calculate the dry matter content, the following procedures were carried out on the storage root harvested to first estimate specific gravity using the gravimetric method.


(1) $$Specific\;gravity=\frac{Weight\;in\;air}{Weight\;in\;air-Weight\;in\;water}.$$

Thereafter, the following formula was applied:


(2) Dry matter content = 158.3 × specific gravity – 142.

##### 2.3.2.2 Starch content (SC)

The percentage content of starch was determined using the following formula:


(3) Root starch content = 210.8 × specific gravity − 213.4.

##### 2.3.2.3 HI

It is the ratio of the total storage root weight and the total weight of the plant (stem, stump, and storage root weight).


(4) $$HI=\;\frac{Total\;storage\;root\;weight}{Stem\;weight+stump\;weight+storage\;root\;weight},$$

$$HI=\;\frac{Root\;weight}{\left(Root\;weight+Biomass\right)}.$$

##### 2.3.2.4 Root density

This is a derivative measurement that is a function of the ratio of total root weight (kg) and the volume of storage root system (m^3^).


(5) $$\begin{array}{c} Root\;density\\ =\;Total\;root\;weight/Volume\;of\;the\;storage\;root\;system, \end{array}$$

where *Volume of the storage root system* is the *root system length* × *root system width* × *root system depth*.

##### 2.3.2.5 Dry yield

This is derived using the following formula:


(6) $$Dry\;yield=\frac{FRYD\times DMC}{100}.$$

##### 2.3.2.6 Fresh root yield (ton/ha)

This is derived using the following formula:


(7) $$FRYD=\frac{Root\;weight}{Area\;of\;plot\;harvested\left(m^{2}\right)}\times \frac{10,000}{1,000}\;\mathrm{OR}$$

$$FRYD=\frac{No.\;of\;hvtd\;plt\;per\;plot\;\times Avrge\;of\;rtwt\;per\;plt\;per\;plot}{Plot\;area\;\left(m^{2}\right)}\times 10.$$

##### 2.3.2.7 Root size area:

This trait is derived by multiplying the root length and breadth of selected three roots as biggest, moderate, and smallest and calculating the average of each area.


(8) Root size area = Root length × root breadth.

##### 2.3.2.8 Index of root formation and bulking (FBI)

This is a multivariate trait, derived using selection index methods, which combined the traits that were significantly correlating with yield. The formula used was


(9) $$FBI=\sum_{i=1}^{n}\left(\beta_{n}X_{n}\right),$$

where *FBI* is the index of root formation and bulking, *β_n_* is the weight of the different independent variables observed, and *X_n_* indicates the different independent variables (fresh root yield, dry matter content, root density, root size area, and number of storage roots).

### 2.4 Statistical analysis and model

In order to account for the variance components, an analysis of variance was carried out for all the traits. Statistics were analyzed using the lme4 package in R. In order to analyze phenotypic data, the mixed model was fitted (Bates et al., 2014), taking into account the lmer function of the lme4 r package.


(10) $${\text{Y}}_{\text{ijkl}}=\text{μ}+{\text{g}}_{\text{i}}+{\text{τ}}_{\text{j}}+{\text{δ}}_{\text{k}}+{\text{ρ}}_{\text{ij}}+{\text{σ}}_{\text{ik}}+{\text{φ}}_{\text{jk}}+{\text{ω}}_{\text{ijk}}+{\left({\text{β}}_{\text{l}}\right)}_{\text{ijk}}+{\text{ε}}_{\text{ijkl}},$$

where Y_ijkl_ is the phenotypic performance of ith genotype at the jth harvesting time and kth environment; μ is the total mean; $g_{i}$ is the effect of the ith genotype; $\tau_{j}$ is the effect of the jth harvesting time; $\delta_{k}$ is the effect of the kth environment; $\rho_{ij}$ is the effect of the interaction between the ith genotype and the jth harvesting time; $\sigma_{ik}$ is the effect of the interaction between the ith genotype and the kth environment; $\varphi_{jk}$ is the interaction effect between the jth harvesting time and the kth environment; $\omega_{ijk}$ is the effect of the interaction between the ith genotype and the jth harvesting time in the kth environment; ${\left(\beta_{l}\right)}_{ijk}$ is the effect of the lth block within the ith genotypes, jth harvesting time, and kth environment; and $\varepsilon_{ijkl}$ is a random error.

Other meta-analyses, such as principal component analysis (PCA), diversity analysis, association analyses between early root storage formation and bulking attributes, and gene annotation, were carried out using the r packages and genomic website National Center for Biotechnology Information (NCBI). Boxplots were also used in the visualization analysis to provide the data summary and provide a clearer representation of the variability and distribution within the data.

### 2.5 DNA extraction

The method of DNA extraction was adopted from Rajahmundry (2008), where for each genotype, a polythene sampling bag was used to collect a total of 5 mg of young leaf tissue, which was then chilled with ice. A water bath at 65°C was used to create the extraction buffer, which contained 200 mM Tris-HCl, 50 mM ethylenediaminetetraacetic acid (EDTA), 2 M NaCl, 2% cetyltrimethylammonium bromide (CTAB), and 3% mercaptoethanol. Leaf tissue samples were ground for 5 minutes in liquid nitrogen using sterile 4-mm stainless steel ball bearings in a FastPrep-24TM, 5G tissue homogenizer. The ground samples were added to 1 mL of prewarmed (65°C) CTAB buffer (200 mM Tris-Cl, 50 mM EDTA, 2 M NaCl, 2% CTAB, and 3% mercaptoethanol) and vortexed at 3,000 rpm for 30 s to yield high-molecular-weight DNA. During incubation, tubes were gently stirred every 10 minutes while being heated in a water bath for 30 minutes at 65°C; 500 L of chloroform:isoamyl alcohol (24:1) was added, and the samples were then mixed by inverting the tubes 20 to 30 times. The top layer was then recovered into a fresh tube after 15 minutes of centrifuging the samples at 15,000 rpm. To guarantee the integrity of the isolated DNA, this procedure—chloroform:isoamyl alcohol—was repeated. With 1/5 volume of 5 M NaOAC and 2.5 volume of cold, absolute ethanol (stored at or below 20°C), DNA was precipitated. After a gentle inversion to mix the samples, they were incubated at 20°C for 60 minutes. After centrifuging the samples, the supernatant was decanted to recover the DNA pellet. The DNA pellet was twice washed in 500 L of cold (20°C), 70% (v/v) ethanol before being allowed to air-dry; 400 mg of RNase-A was added to 100 L of low-EDAT TE buffer (1 mM Tris-Cl and 0.1 mM EDTA) containing the DNA pellet. A NanoDrop spectrophotometer was used to determine the purity and concentration of the DNA.

### 2.6 Genotyping, SNP calling, and haplotype estimation

For genotyping using the DArTseq technology, DNA samples were sent to the Integrated Genotyping Service and Support (IGSS) at the Biosciences Eastern and Central Africa-International Livestock Research Institute (BecA-ILRI) Hub. The GBSapp pipeline was used to pre-process the fastq files, call variants and dosages, and perform variant filtering. The pipeline incorporates a variety of programs, such as GATK v3.7 (Zhu et al., 2014), which was designed to work best with highly heterozygous and polyploid species (Wadl et al., 2018).

Using 71,585 DArT markers, whole-genome genotyping of 220 cassava clones was carried out on the platform developed by Cruz et al. (2013). The marker order and position were deduced from a consensus DArT map and used to integrate the markers into a linkage map. The mean polymorphic information content ranged from 0.0 to 0.50, with a repeatability index of 0.93.

In accordance with Mwadzingeni et al. (2017), problematic SNPs with more than 5% of missing data were filtered out of the DArTseq SNP-generated markers using imputation. To genotype each individual, 71,585 SilicoDArT markers spread over 18 chromosomes were used. In the analysis, 68,383 DArTseq markers were used after the missing values were imputed.

### 2.7 Genetic relationship, population structure, and linkage disequilibrium

After removing biased batch-specific markers, 68,383 polymorphic SNP markers were left for use in the genetic relationship and population structure analyses. The polymorphic SNP markers were employed for the analysis of the population structure and genetic relationships based on a cut-off of 0.85.

Using the set of 68,383 SNP markers selected, linkage disequilibrium analysis was carried out through Genome Association and Prediction Integrated Tool (GAPIT) serial packages (Lipka et al., 2012) and implemented in the R-software v3.5.1. Only *p*-values of 0.01 for each pair of loci were considered significant, and linkage disequilibrium (LD) was calculated as squared allele frequency correlations (R^2^). For an LD-based analysis of genome-wide associations, the LD decays were also estimated.

### 2.8 Genome-wide association studies

A total of 71,585 SNP markers were examined in the genome-wide for the selection of polymorphisms. Of these, 2,772 SNPs (3.87%) were excluded because of a minor allele frequency (MAF) below 0.05. The remaining 68,383 polymorphic SNPs with MAF greater than 5% were used for this study.

The population structure (Q) and kinship (K) matrix was estimated to reduce false-positive rates and maximize statistical power. The kinship or relatedness (K) matrix was utilized as a random effect in an enhanced version of the fixed and random model circulating probability unification (BLINK) in order to take population structure into account and minimize false correlations. GAPIT version 3 was used to conduct the analysis in R software v3.5.2 (Lipka et al., 2012). The VanRaden method (VanRaden, 2008) was used to calculate the variance–covariance kinship matrix (K). To evaluate the genetic diversity within the collection (N = 220), GAPIT automatically generated the first three principal components of the dataset. The genome-wide association study (GWAS) model took into account the first three major SNP data components. The Li et al. (2013) approach was used to calculate the Bonferroni threshold for *p*-values based on the number of markers (*p* = 1/n, n = total SNP used).

### 2.9 Identification of candidate genes

The position of the highly significant SNP markers was explored by subjecting them to fine mapping and BLAST search on NCBI Genome Viewer v6.0 to annotate genomic regions and detect the nearby putative candidate genes associated with storage root formation and bulking. Putative genes within the significant SNP region were searched with respect to the significant SNP positions flanking right and left. Using the databases of the European Molecular Biology Laboratory-European Bioinformatics Institute (EMBL-EBI) and Universal Protein Resource (UniProt), the functions of the genes linked to the detected SNPs were found (Cingolani et al., 2012).

## 3 Results

### 3.1 Phenotypic variations and correlations

**Figure 3:**
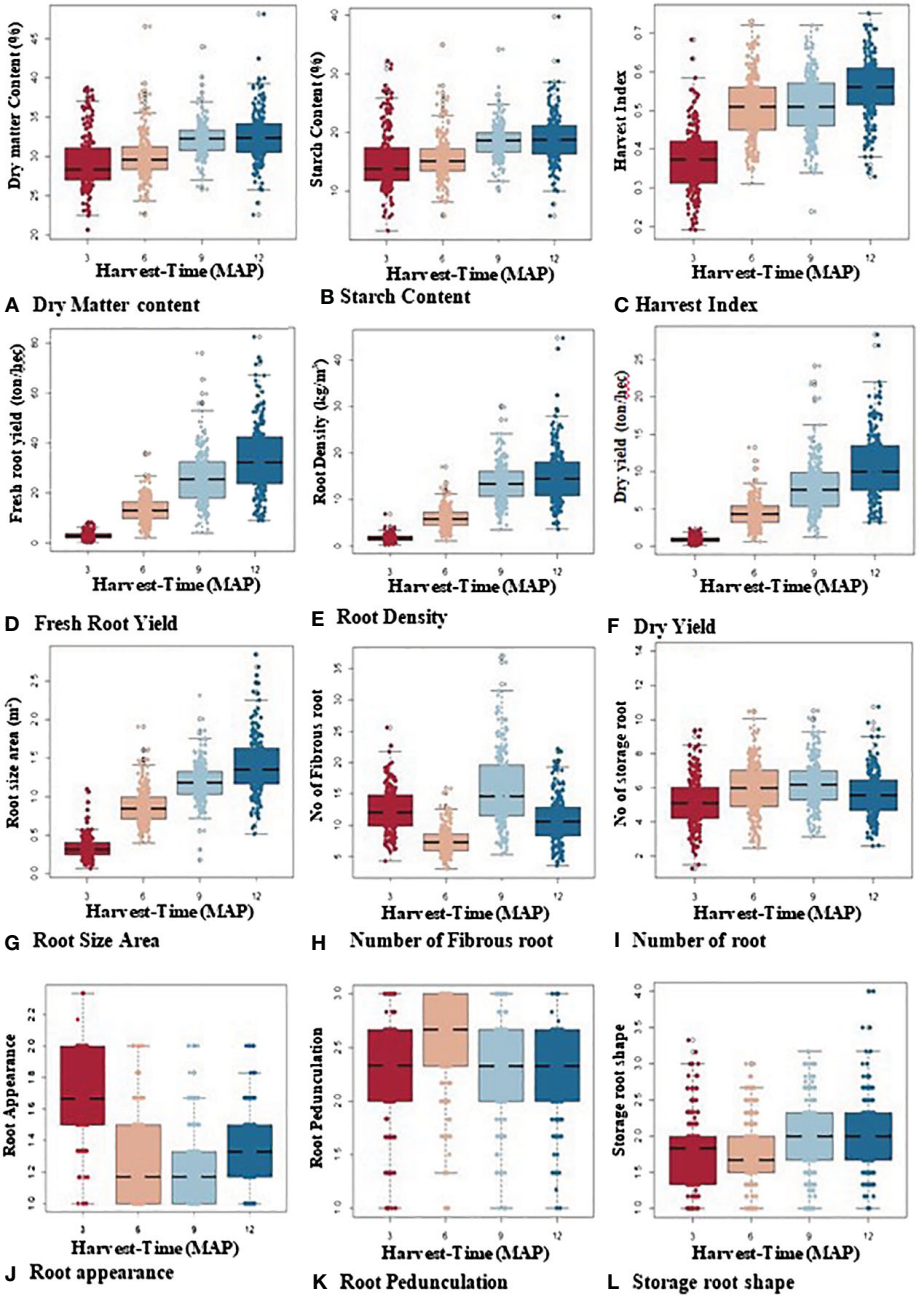
Boxplot showing overall variability and dispersion of bulking traits over four harvest times (3, 6, 9, and 12 MAP).

A boxplot was used to visualize the distribution and kinetic pattern of genotype performances across the various plant ages of 3, 6, 9, and 12 MAP (**Figure 3**). The findings show that every trait was subject to a normal distribution at each stage of plant development. With a progressive increase from 3 to 9 MAP, the starch content and dry matter content, with mean ranges of 21%–49% and 2%–40%, respectively, exhibited a similar trend. However, at 9 to 12 MAP, they showed no change.

With a wider distribution of data at 9 and 12 MAP in the bulking process, fresh storage root yield (4–81 t/ha), root density (2–44 kg/m^3^), dry yield (1–11 t/ha), harvest index (0.2–0.75), and storage root size area (0.15–2.90 cm^2^) all increased over time. Although alterations from 6 and 9 MAP were more gradual, the number of storage roots exhibited a similar pattern. The skin color appearance of the roots varied according to the plant’s age. At 3 MAP, the majority of the storage roots were gray (2-scale = gray color), turned dark brown (1-scale = dark brown color), and then primarily turned light brown (1.5-scale = light brown color) at 6 and 9 MAP. Similar patterns were seen in root pedunculation (1-scale = presence of peduncle, 2-scale = sessile, and 3-scale = no peduncle), though variations at 6 MAP were more gradual and abruptly dropped to sessile at 9 and 12 MAP. At the early stages of bulking (3 to 6 MAP), root shape varied, but for the majority of genotypes, it was unaffected at 9 and 12 MAP.

For genotypes assessed for the various plant ages, phenotypic trait correlation analysis was conducted independently (**Table 1**). The results of the correlation at 3 MAP showed strong positive and significant correlations (*p*< 0.001) observed between the bulking index and the different bulking traits like dry yield (0.76), fresh root yield (0.75), root density (0.77), number of storage roots (0.63), storage root size area (0.47), and harvest index (0.43) but showed slightly positive and significant correlations (*p*< 0.05) with dry matter content (0.24), starch content (0.18), and shape (0.16). The bulking index at 3 MAP did not significantly correlate (*p* > 0.05) with skin root color appearance, pulp color, number of fibrous roots, or root pedunculation.

**Table 1:** Pearson’s correlation of the phenotypic traits measured at 3, 6, 9, and 12 MAP and their bulking indices.

3 MAP														
**B_Index_3**														
−0.02	**App**													
−0.07	0.11	**Pulp_Col**												
0.24*	−0.1	0.06	**DMC**											
0.76***	−0.04	−0.12	0.05	**DRYD**										
0.01	−0.03	−0.15	−0.14	0.03	**FibRt**									
0.75***	0.01	−0.11	−0.19*	0.97***	0.06	**FRYD**								
0.43**	0.01	−0.04	0.13	0.39**	−0.15	0.36**	**HI**							
0.02	0.05	0.09	−0.02	−0.07	−0.06	−0.07	0.09	**Pend**						
0.77***	0.01	−0.1	−0.15	0.58***	−0.03	0.62***	0.42**	0.05	**rt_density**					
0.47**	0.09	0.06	−0.19*	0.25*	−0.02	0.31**	0.08	0.04	0.27*	**rt_size**				
0.63***	−0.01	−0.11	−0.20	0.43**	0.23*	0.48**	0.18*	0.08	0.51***	0.22*	**Rtno**			
0.18*	−0.1	0.03	0.95***	−0.08	−0.17	−0.22*	0.11	−0.07	−0.19*	−0.20	−0.21*	**SC**		
−0.16*	0.12	−0.09	−0.09	−0.11	−0.04	−0.09	−0.09	−0.26*	−0.11	0.01	−0.15	−0.02	**Shape**	
6 MAP														
**B_Index_6**														
−0.05	**App**													
0.01	0.18	**Pulp_Col**												
0.94***	−0.04	0.01	**DMC**											
0.32*	−0.10	−0.07	0.18*	**DRYD**										
−0.06	−0.03	−0.05	−0.07	−0.02	**FibRt**									
0.24*	−0.06	0.01	−0.08	0.45**	−0.01	**FRYD**								
0.05	−0.10	−0.01	−0.02	0.09	−0.05	0.21*	**HI**							
0.15	0.04	0.07	0.07	0.11	−0.08	0.23*	0.09	**Pend**						
0.27*	0.01	−0.10	−0.10	0.32*	0.06	0.72***	0.26*	0.20	**rt_density**					
0.15	−0.05	0.01	−0.06	0.24*	−0.02	0.62***	0.10	0.20	0.53***	**rt_size**				
0.18*	0.03	−0.10	0.01	0.23*	0.24*	0.42**	0.06	0.10	0.54***	0.22*	**Rtno**			
0.94***	−0.04	0.01	0.92***	0.18*	−0.07	−0.08	−0.02	0.07	0.00	−0.06	0.01	**SC**		
−0.04	0.06	−0.14	−0.03	0.01	0.17	−0.01	0.01	−0.15	−0.06	−0.03	−0.06	−0.03	**Shape**	
9 MAP														
**B_Index_9**														
−0.02	**App**													
−0.14	0.16	**Pulp_Col**												
0.19*	−0.1	−0.15	**DMC**											
0.9	−0.02	−0.18*	0.17	**DRYD**										
−0.03	−0.06	−0.01	−0.21*	−0.05	**FibRt**									
0.97***	0.01	−0.12	0.04	0.91***	0.01	**FRYD**								
0.38**	0.04	−0.07	0.10	0.40**	−0.05	0.35**	**HI**							
0.14	−0.08	−0.07	−0.04	0.05	−0.08	0.12	0.01	**Pend**						
0.03	0.03	−0.14	0.07	0.14	−0.05	0.16*	−0.02	−0.06	**Rot_no**					
0.78***	0.01	−0.13	−0.16	0.64***	−0.04	0.73***	0.33*	0.22*	0.05	**rt_density**				
0.26*	−0.08	−0.17	0.00	0.21*	−0.03	0.24*	0.12	0.06	0.07	0.35**	**rt_size**			
0.40	0.02	−0.01	−0.13	0.34*	0.17	0.41**	0.04	0.05	0.23*	0.36**	0.04	**Rtno**		
0.18*	−0.10	−0.16	0.98***	0.17	−0.22*	0.04	0.10	−0.05	0.08	−0.16	−0.01	−0.13	**SC**	
0.07	0.07	0.03	0.02	0.10	−0.04	0.09	0.01	−0.11	0.09	0.02	0.07	0.06	0.03	**Shape**
12 MAP														
**B_Index_12**														
0.06	**App**													
−0.02	0.12	**Pulp_Col**												
0.06	0.04	−0.15	**DMC**											
0.94***	0.05	−0.01	0.15	**DRYD**										
−0.02	−0.06	−0.05	0.08	−0.03	**FibRt**									
0.97***	0.05	0.02	−0.08	0.94***	−0.05	**FRYD**								
0.33*	−0.11	−0.03	0.09	0.32*	0.01	0.30*	**HI**							
−0.03	−0.01	0.13	−0.14	−0.02	−0.01	−0.02	−0.06	**Pend**						
−0.07	−0.04	−0.05	−0.04	−0.02	0.07	−0.02	0.02	0.11	**Rot_no**					
0.76***	0.02	−0.06	−0.09	0.58***	0.04	0.65***	0.29*	0.06	0.07	**rt_density**				
0.40**	0.07	0.08	−0.16	0.36**	0.00	0.41**	−0.03	0.18*	0.01	0.28*	**rt_size**			
0.49**	0.06	−0.13	−0.09	0.45**	0.15	0.47**	0.12	0.06	−0.03	0.35**	0.26*	**Rtno**		
0.06	0.04	−0.15	0.98***	0.15	0.08	−0.08	0.09	−0.14	−0.04	−0.09	−0.16	−0.1	**SC**	
0.01	0.04	0.09	−0.06	−0.01	0.11	−0.02	−0.11	−0.09	−0.1	0.03	−0.01	0.11	−0.06	**Shape**
Bulking Indices														
**B_Index_3**														
0.03				**B_Index_6**										
0.16				−0.02				**B_Index_9**						
0.25				−0.05				0.36				**B_Index_12**		

The correlation level is color-coded according to the color key plotted. Correlations with *, **, and *** were significant at 0.05, 0.01, and 0.001 levels, respectively. B_Index_3, _6, _9, and _12 mean bulking index at 3, 6, 9, and 12 MAP, respectively.

App, appearance of storage root; Pulp_Col, pulp color; DMC, dry matter content; DRYD, dry root yield; FibRt, number of fibrous roots; FRYD, fresh root yield; HI, harvest index; Pend, pedunculation; Rot_no, number of storage root rot; rt_density, root density; rt_size, storage root size; Rtno, number of storage root; SC, starch content; Shape, storage root shape.

At 6 MAP, FBI showed a significant but weak correlation (*p*< 0.05) with dry yield (0.32), FRYD (0.24), root density (0.27), number of roots (0.18), and root size area (0.15), as well as a very strong positive and significant correlation (*p*< 0.001) with both dry matter content and starch content. Additionally, there was no significant association (*p* > 0.05) between HI at 6 MAP and the root color appearance, pulp color, and number of fibrous roots.

The bulking index at 9 and 12 MAP showed the same pattern of correlation, with a strong positive relationship (*p*< 0.001) with dry yield (0.90 and 0.94, respectively), FRYD (0.97 for both), and root density (0.78 and 0.76, respectively), as well as a moderate relationship with HI (0.39 and 0.33, respectively), number of storage roots (0.40 and 0.49, respectively), and storage root size area (0.26 and 0.40, respectively). At 12 MAP, the bulking index revealed no significant association (*p* > 0.05) between the starch content and dry matter content; however, at 9 MAP, it indicated a positive significant and weak correlation (*p*< 0.05) of 0.18 and 0.19, respectively.

With the exception of the positive significant correlation (*p*< 0.01) of 0.36 between the bulking index at 12 MAP and bulking index at 9 MAP, there were little to no correlations between the bulking index of the four harvest times (3, 6, 9, and 12 MAP).

Across the harvest time, the bulking index recorded no significant correlation (*p* > 0.05) with root color appearance, pulp color, number of fibrous roots, and root pedunculation.

The analysis of variance (ANOVA) results at various plant ages are shown in **Table 2**. For the majority of the analyzed variables, the genotypic variance was significant (*p*< 0.001), indicating high phenotypic variance for all the traits: appearance (app), root shape, root density (rt_density), root size (rt_size), number of storage root (rtno), DMC, SC, fresh root yield (FRYD), HI, and dry root yield (DRYD). The results also showed that the influence of genotype by environment interactions is substantial. At 3 MAP, all traits showed strong broad-sense heritabilities ranging from 0.55% to 0.68%, with high significant (*p*< 0.001) genotypic variance and interaction variance. At 6 MAP, every trait had genotypic variance that was highly significant (*p*< 0.001) and also the interaction variance. The traits ranged from a moderate level to a high level of broad-sense heritability (H^2^) of 0.31% to 0.66%. For all the phenotypes at 9 and 12 MAP, there were also significant (*p*< 0.001) genotypic variance and interaction variance, with a moderate to high range of broad-sense heritability of 0.52%–0.68% and 0.28%–0.66%, respectively.

**Table 2:** Estimation of mean, range, and variance components and broad-sense heritability of phenotypic traits evaluated at 3, 6, 9, and 12 MAP.

Trait	Mean (range)	$\sigma_{G}^{2}$	$\sigma_{G\times E}^{2}$	$\sigma_{e}^{2}$	H^2^	LSD_0.05_
3 MAP						
App	1.29 (1.00–2.33)	0.42***	0.32***	0.04	0.68	0.21
Shape	1.78 (1.00–3.33)	1.53***	0.93***	0.41	0.64	0.73
rt_density	5.82 (0.21–6.94)	6.01***	5.43***	2.23	0.55	1.69
rt_size	0.87 (0.07–1.10)	0.13***	0.11***	0.05	0.55	0.25
Rtno	5.94 (1.24–14.00)	15.50***	8.11***	5.50	0.62	0.76
DMC	32.06 (20.65–38.00)	54.47***	39.52***	21.19	0.57	0.21
SC	18.36 (3.20–29.00)	95.45***	69.53***	37.47	0.57	6.94
FRYD	13.19 (0.21–8.43)	14.78***	8.75***	4.47	0.63	2.40
HI	0.56 (0.19–0.68)	0.05***	0.03***	0.01	0.67	0.14
DRYD	4.27 (0.06–2.43)	1.21***	0.70***	0.37	0.63	0.69
6 MAP						
App	1.29 (1.00–2.00)	0.43***	0.29***	0.08	0.66	0.32
Shape	1.78 (1.00–3.00)	0.38***	1.11***	0.28	0.31	0.60
rt_density	5.82 (91.00–15.00)	31.64***	22.50***	11.88	0.58	3.91
rt_size	0.87 (0.40–1.80)	0.34***	0.19***	0.13	0.60	0.41
Rtno	5.94 (2.60–11.00)	14.01***	9.48***	5.67	0.57	2.70
DMC	32.06 (26.00–37.00)	20.75***	18.98***	10.34	0.51	3.65
SC	18.36 (11.00–25.00)	37.77***	33.33***	18.52	0.52	4.88
FRYD	13.19 (2.20–36.00)	178.95***	130.94***	69.26	0.57	9.44
HI	0.56 (0.30–0.70)	0.04***	0.03***	0.02	0.53	0.15
DRYD	4.27 (0.60–12.00)	18.76***	13.80***	7.24	0.57	3.05
9 MAP						
App	1.27 (1.00–2.00)	0.44***	0.29***	0.06	0.68	0.28
Shape	1.97 (1.00–4.00)	1.83***	1.32***	0.24	0.67	0.56
rt_density	13.69 (3.10–42.00)	119.84***	77.63***	48.80	0.58	7.93
rt_size	0.19 (0.10–2.70)	0.06***	0.04**	0.03	0.55	0.20
Rtno	5.60 (2.80–11.00)	12.05***	8.95***	6.55	0.52	2.91
DMC	29.80 (23.00–41.00)	41.39***	25.13***	13.11	0.62	4.11
SC	15.39 (5.80–28.00)	73.60***	44.63***	23.00	0.62	5.44
FRYD	26.13 (3.40–82.00)	753.40***	469.60***	222.30	0.62	16.92
HI	0.51 (0.20–0.80)	0.04***	0.03***	0.02	0.53	0.14
DRYD	7.82 (0.90–27.00)	77.75***	44.35***	21.49	0.64	5.26
12 MAP						
App	1.32 (1.00–2.00)	0.45***	0.30***	0.08	0.66	0.32
Shape	2.00 (1.00–4.00)	0.48***	1.16***	0.67	0.28	0.93
rt_density	14.76 (2.70–48.00)	222.60***	187.50**	144.50	0.48	13.64
rt_size	1.57 (0.40–2.70)	0.92***	0.79***	0.44	0.52	0.75
Rtno	5.63 (2.00–10.00)	10.95***	8.89***	5.10	0.53	2.56
DMC	31.37 (23.00–41.00)	48.90***	29.01***	16.93	0.61	4.67
SC	18.80 (5.80–30.00)	83.93***	51.88***	28.58	0.61	6.07
FRYD	32.57 (5.50–75.00)	973.10***	721.10***	393.30	0.56	22.51
HI	0.57 (0.30–0.80)	0.04***	0.03***	0.02	0.53	0.16
DRYD	10.24 (1.70–23.00)	97.03***	74.25***	39.60	0.56	7.14
Bulking indices						
B_index_3	3.65 (3.37–3.86)	1.05***	0.72***	0.45	0.56	0.75
B_index_6	5.67 (5.10–6.37)	4.81***	3.58***	1.82	0.57	1.53
B_index_9	7.56 (6.41–9.99)	16.81***	10.44***	4.99	0.62	2.54
B_index_12	8.74 (7.72–10.14)	22.87***	17.34***	9.82	0.55	3.56

*, **, and *** indicate significance at p< 0.05, p< 0.01, and p< 0.001, respectively. $\sigma_{G}^{2}$ , $\sigma_{G\times E}^{2}$ , and $\sigma_{e}^{2}$ represent genotypic, genotype × environment interactions, and error variance, respectively. B_Index_3, _6, _9, and _12 mean bulking index at 3, 6, 9, and 12 MAP.

H^2^, broad-sense heritability; LSD, least significant difference; App, appearance of storage root; DMC, dry matter content; DRYD, dry root yield; FRYD, fresh root yield; HI, harvest index; rt_density, root density; rt_size, storage root size; Rtno, number of storage root; SC, starch content; Shape, storage root shape; B_Index_3, _6, _9, and _12 means Bulking index at 3, 6, 9, and 12 MAP.

Except for the bulking index at 3 MAP, which revealed no significant difference for genotypic variance, all bulking indices indicated significant differences for genotypic variance and the interaction of genotype by environment.

### 3.2 Genetic relationship, population structure, and linkage disequilibrium

#### 3.2.1 Analysis of SNP markers

**Supplementary Figure S1** shows the SNP marker coverage throughout the 18 chromosomes. A total of 68,383 polymorphic SNP markers were found and used in this study after being initially checked for chip quality and missing data. After removing SNP markers with a high missing rate of MAF lower than 5%, this number passed quality control checks and was submitted as DArTseq SNP markers. With a maximum of 8,307 markers, Chromosome 1 had the highest marker density, followed by Chromosome 14 with 5,544 markers, and Chromosome 7 had the lowest marker density with 2,088 markers.

#### 3.2.2 Population structure

**Figure 4:**
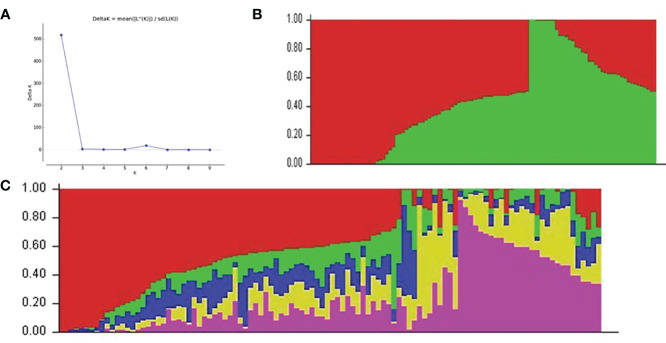
The five subpopulations of the 212 cassava cultivars using SNP markers. **(A)** Best delta K estimation. **(B)** Estimated population structure of 212 cassava cultivars as revealed by SNP markers for K = 2 and K = 5 **(C)**. Red, purple, green, blue, and yellow colors represent subpopulations 1, 2, 3, and 4, respectively. SNP, single-nucleotide polymorphism.

Two hundred twenty cultivars were originally obtained from the CIAT, IITA, local farmers, NextGen breeding project, and NRCRI. Apart from six landraces, the majority of the set of germplasms were modern cultivars. In the population structure analysis, the K probability value of 5 was the most likely portion of the population that had the highest value of In P(D) and was consistent with the significant delta K value = 2, as the best delta K estimation (**Figure 4A**). The estimated population structure of the 212 cassava cultivars as revealed by SNP markers for K = 2 is shown in **Figure 4B**. The estimate at K = 5 clearly showed the best subpopulations represented as red, purple, green, blue, and yellow (**Figure 4C**), which was well supported by the VanRaden kinship algorithm (**Supplementary Figure S2**). All cultivars were classified into five subgroups, which are generally in accordance with the sources of germplasm.

PCA was used to summarize the genetic variation in the cultivars. Based on the principal component analyses, all the cassava cultivars were broadly categorized into five groups—category one (96), category two (62), category three (34), category four (22), and category five (6)—with respect to the displayed plot in **Supplementary Figure S4**. The PC1 and PC2 accounted for 35% and 21% of genotypic variability, respectively. This indicates a moderate level of genetic diversity in the *M. esculenta* germplasm used in the study.

#### 3.2.3 Linkage disequilibrium

**Figure 5:**
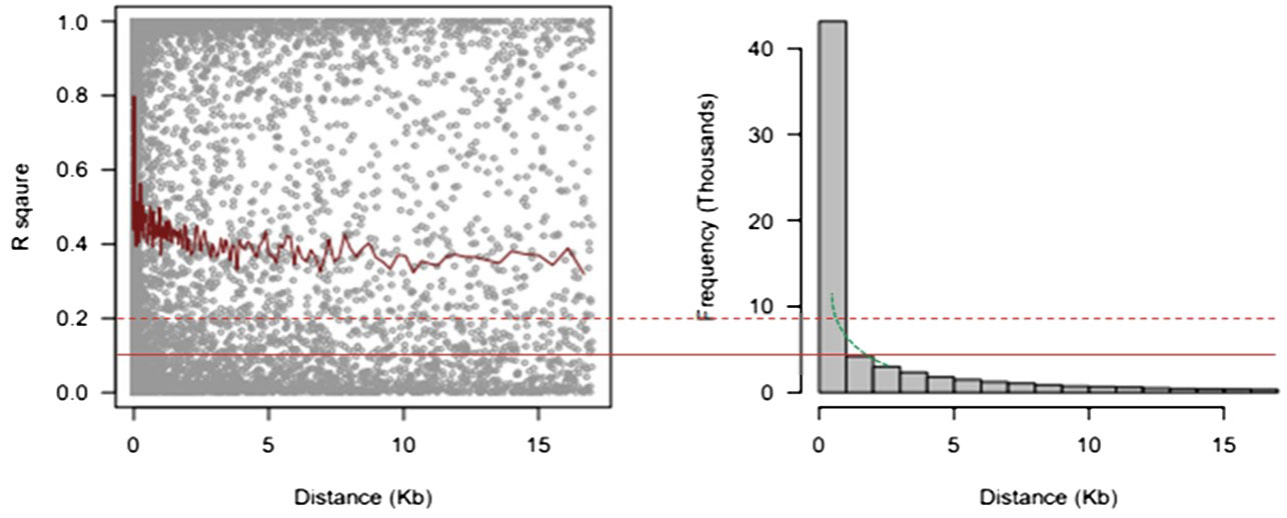
9 Linkage disequilibrium decay measured as r^2^ against the genetic distance between pairs of SNPs. SNPs, single-nucleotide polymorphisms.

A total of 68,813 SNPs were used to evaluate the whole genome’s LD with MAF > 0.05. The LD (r^2^) between adjacent pairs of markers was plotted against the distance in kb to represent the genome-wide LD decay (**Figure 5**). According to the results, LD degraded differently depending on the physical distance. With increasing physical distance, a sharp decline in LD was seen. The average physical distance was 1.50 kb and 0.65 kb using cut-offs of r^2^ = 0.1 and 0.2, respectively.

### 3.3 Genome-wide association study results

**Figure 6:**
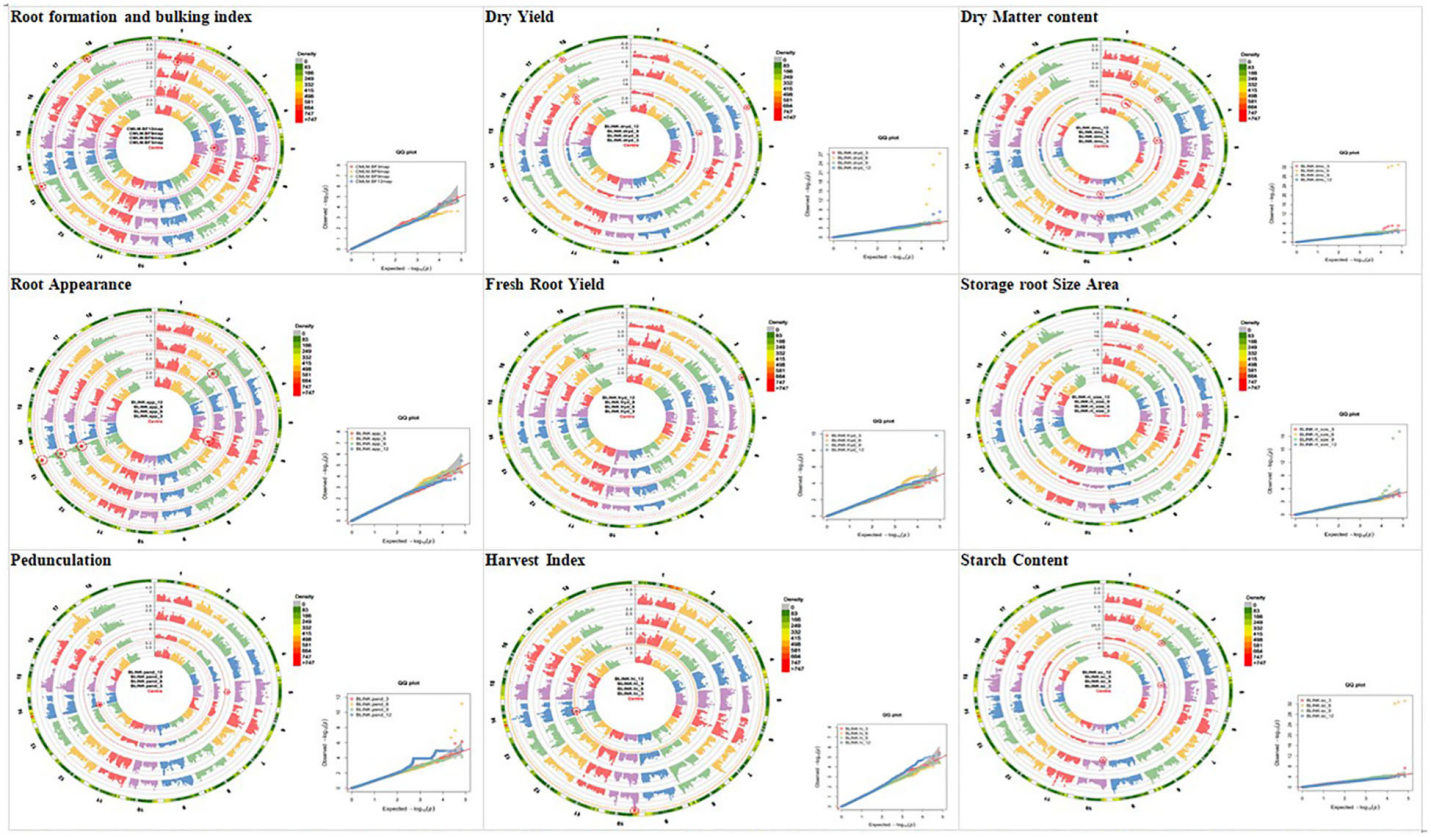
Circular-Manhattan plots and quantile–quantile plots for SNP significantly associated with bulking traits and the bulking index at 3, 6, 9, and 12 MAP identified by genome-wide association study based on the enhanced version of fixed and random model circulating probability unification (BLINK). SNP, single-nucleotide polymorphism.

Circular-Manhattan plots and quantile–quantile (Q-Q) plots were used to visualize SNPs that are significantly associated with bulking traits and the bulking index at 3, 6, 9, and 12 MAP, based on the enhanced version of BLINK. The y-axis of the Manhattan plots describes the negative log base 10 of the *p*-value for each of the SNP markers in the genome, and the x-axis in circular shape shows the chromosome numbers. The red threshold lines show genome-wide significance (*p*-value<6 × 10^−7^). Each dot represents a SNP arranged across the chromosomes from left to right, while the height of the dot dispersion corresponds to the strength of association to traits at 3, 6, 9, and 12 MAP (**Figure 6**). In this study, significant peaks above the threshold were observed across the different plant ages. The Q-Q plots of the bulking traits revealed a close distribution of observed associations (*p* values) to the distribution of the expected associations. This simply means that the GWAS methods used have control for spurious association.

The significant SNP markers were summarized in **Table 3**, where a total of 45 SNP markers were recorded for the traits across the four plant ages, out of which nine SNP markers were recorded for 3 MAP, 17 were recorded for 6 MAP, seven were recorded for 9 MAP, and 12 were recorded for 12 MAP.

**Table 3:** Total number of the significant SNPs across plant age.

Plant age	Bulking index	Root formation traits	Bulking traits	Total SNP/plant age
3 MAP	1	2	6	9
6 MAP	0	6	11	17
9 MAP	2	1	3	7
12 MAP	2	1	4	12
Total SNP/trait type	5	10	24	45

SNP, single-nucleotide polymorphism.

A total of 45 SNPs passed the Bonferroni significance threshold, as shown in **Table 4**. GWAS identified 10 significant SNP markers for the root formation traits (root appearance, six SNPs; root pedunculation, four SNPs), 24 significant SNP markers for the bulking traits (DMC, seven SNPs; DRY, six SNPs; FRYD, two SNPs; HI, two SNPs; storage root size area, three SNPs; SC, four SNPs). No significant SNPs were observed for root density and number of storage roots. The genetic/allelic effect unit of any single SNP to the phenotypic variation was estimated across the traits (**Table 4**).

**Table 4:** List of SNP markers that are significant using BLINK model.

Traits	MAP	SNP markers	Chr	Position	*p*-Value	MAF	Allelic effect
Index of root bulking and formation							
Root bulking and formation index	3	S5_13850266	5	13850266	1.11E−05	0.160464	−29.1499
Root bulking and formation index	9	S5_26556768	5	26556768	2.90E−05	0.103019	25.39229
Root bulking and formation index	9	S1_20402446	1	20402446	3.05E−05	0.121226	28.38468
Root bulking and formation index	12	S18_3832020	18	3832020	2.34E−05	0.172171	13.54253
Root bulking and formation index	12	S18_3834291	18	3834291	2.47E−05	0.169811	13.59251
Root formation traits							
Root color appearance	3	S6_21635414	6	21635414	4.45E−05	0.226415	0.12703
Root color appearance	6	S3_4741499	3	4741499	1.54E−05	0.306604	0.108654
Root color appearance	6	S3_4741478	3	4741478	3.13E−05	0.325472	0.106152
Root color appearance	6	S13_25995725	13	25995725	3.72E−05	0.160377	−0.13317
Root color appearance	9	S13_25995725	13	25995725	1.48E−06	0.160377	−0.15897
Root color appearance	12	S13_25995725	13	25995725	4.02E−06	0.160377	−0.1504
Root pedunculation	3	S14_1671178	14	1671178	7.11E−07	0.351415	−0.25682
Root pedunculation	6	S17_15171469	17	15171469	7.06E−12	0.054151	−1.1589
Root pedunculation	6	S5_15110157	5	15110157	2.50E−08	0.488208	−0.21639
Root pedunculation	6	S16_24656274	16	24656274	2.23E−07	0.109434	−0.99412
Root bulking traits							
Dry matter content	3	S5_1557006	5	1557006	9.53E−08	0.346698	−1.67951
Dry matter content	3	S10_2912754	10	2912754	1.13E−07	0.075472	−3.36028
Dry matter content	3	S2_5069109	2	5069109	1.93E−07	0.120283	−2.65119
Dry matter content	3	S2_10059232	2	10059232	5.13E−07	0.478774	1.629352
Dry matter content	6	S10_2319500	10	2319500	1.10E−33	0.111792	356.0528
Dry matter content	6	S2_1937678	2	1937678	2.90E−33	0.107075	−357.556
Dry matter content	6	S3_3324735	3	3324735	1.22E−32	0.102358	−354.827
Dry yield	6	S6_19749539	6	19749539	5.39E−28	0.104717	73.51314
Dry yield	6	S17_18894518	17	18894518	2.91E−24	0.092541	−37.7513
Dry yield	6	S17_13553658	17	13553658	1.68E−16	0.070635	33.72815
Dry yield	6	S4_26245979	4	26245979	1.99E−11	0.074174	−33.4205
Dry yield	12	S4_8840623	4	8840623	5.29E−09	0.320755	2.041234
Dry yield	12	S18_3834291	18	3834291	3.37E−08	0.169811	2.551225
Fresh root yield	6	S18_9930952	18	9930952	2.57E−06	0.064861	6.291614
Fresh root yield	12	S4_8840623	4	8840623	1.54E−10	0.320755	7.446926
Harvest index	3	S14_4092696	14	4092696	3.44E−06	0.120283	0.055754
Harvest index	12	S10_2601853	10	2601853	5.54E−06	0.308962	0.040868
Storage root size area	9	S9_26051761	9	26051761	7.55E−20	0.08338	0.612754
Storage root size area	9	S5_12439769	5	12439769	2.93E−18	0.231581	0.635909
Storage root size area	9	S2_4134534	2	4134534	2.60E−07	0.065091	0.123814
Starch content	3	S5_1735523	5	1735523	4.90E−08	0.365566	−2.59038
Starch content	6	S10_2319500	10	2319500	7.04E−34	0.101722	473.745
Starch content	6	S2_1937678	2	1937678	1.88E−33	0.070075	−475.691
Starch content	6	S3_3324735	3	3324735	7.98E−33	0.052380	−472.072

MAF, minor allele frequency; Effect, allele effect; p-value, probability for the mixed linear model; Chr, chromosome; SNP, single-nucleotide polymorphism.

#### 3.3.1 Root formation and bulking index

Five SNP markers were significantly associated with the multi-collinear regression index of root formation and bulking. The significant markers associated with the trait were found on the following chromosomes: SNP S5_13850266 was found on Chromosome 5 at 3 MAP; SNPs S5_26556768 and S1_20402446 were found on Chromosomes 5 and 1, respectively, at 9 MAP, and both SNPs S18_3832020 and S18_3834291 were found on Chromosome 18 at 12 MAP. The top significant SNP marker (S5_13850266) explained −29.15 units of the allelic effect (**Table 4**).

#### 3.3.2 Root formation traits

Ten SNP markers were found to be significantly associated with storage root color appearance and root pedunculation. The significant markers associated with the storage root color appearance were found on the following chromosomes: SNP S6_21635414 on Chromosome 6 at 3 MAP, SNPs S3_4741499 and S3_4741478 on Chromosome 3 at 6 MAP, and S13_25995725 on Chromosome 13 at 6, 9, and 12 MAP. The significant markers associated with the storage root pedunculation were found on the following chromosomes: SNP S14_1671178 on Chromosome 14 at 3 MAP; SNPs S17_15171469, S5_15110157, and S16_24656274 on Chromosomes 17, 5, and 16, respectively. The top significant SNP markers (S13_25995725 and S17_15171469) for the storage root appearance and root pedunculation respectively explained −0.16 and −1.16 units of the allelic effect (**Table 4**).

#### 3.3.3 Root bulking traits

Twenty-four markers displayed significant associations with the root bulking traits. The root bulking traits were characterized by DMC, dry yield, FRYD, HI, storage root size, and starch content. Seven significant SNP markers (four SNPs at 3 MAP and three SNPs at 12 MAP) associated with the dry matter content were found on the following chromosomes: at 3 MAP, SNPs S5_1557006 and S10_2912754 on Chromosomes 5 and 10, respectively, while both SNPs S2_5069109 and S2_10059232 were found on Chromosome 2. At 12 MAP, SNPs S10_2319500, S2_1937678, and S3_3324735 were found on Chromosomes 10, 2, and 3, respectively.

Six significant SNP markers (four SNPs at 6 MAP and two SNPs at 12 MAP) associated with the dry yield were found on the following chromosomes: at 6 MAP, SNPs S6_19749539 and S4_26245979 on Chromosomes 6 and 4, respectively, while both SNPs S17_18894518 and S17_13553658 were found on Chromosome 17. At 12 MAP, SNPs S4_8840623 and S18_3834291 were found on Chromosomes 4 and 18, respectively.

In the same vein, two significant SNP markers (one each at 6 MAP and 12 MAP) were found associated with the fresh root yield: SNPs S18_9930952 and S4_8840623 were respectively on Chromosomes 18 and 4. Two significant SNP markers (one each at 3 MAP and 12 MAP) were also found associated with the harvest index: SNPs S14_4092696 and S10_2601853 were respectively on Chromosomes 14 and 10.

Three significant SNP markers (S9_26051761, S5_12439769, and S2_4134534) were found associated with the storage root size area, and they were all expressed at 9 MAP.

Four significant SNP markers (one SNP at 3 MAP and three SNPs at 6 MAP) associated with the starch content were found on the following chromosomes: at 3 MAP, SNP S5_1735523 was found on Chromosome 5, while at 6 MAP, SNPs S10_2319500, S2_1937678, and S3_3324735 were found on Chromosomes 10, 2, and 3, respectively. The top significant SNP markers (S2_1937678, S6_19749539, S4_8840623, S14_4092696, S5_12439769, and S2_1937678) for the DMC, dry yield, FRYD, HI, storage root size, and starch content, respectively, explained −357.56, 73.51, 7.45, 0.06, 0.64, and −475.69 units of the allelic effect (**Table 4**).

#### 3.3.4 Relationship of the significant SNP marker across age of plant

A Venn diagram of the significant SNP markers across the plant age, traits, and index was carried out to explain the relationship and common markers across the plant age of development (**Supplementary Figure S4**). Four significant SNP markers out of the 48 SNP markers were found to be significantly associated with 6, 9, and 12 MAP categories (**Supplementary Figure S4**). A common locus for 6 and 9 MAP and two common QTLs were detected for 9 and 12 MAP. Similarly, a common locus was detected for 6, 9, and 12 MAP.

Four significant pleiotropic SNP markers were also identified to control more than one trait. Three out of the four SNP markers, i.e., S10_2319500, S2_1937678, and S3_3324735, were significantly associated with starch content and dry matter content, while SNP S4_8840623 was significantly associated with the dry yield, formation, and bulking index (**Supplementary Table S1**).

### 3.4 Candidate gene annotations

The NCBI was used as the reference genome site for finding candidate genes based on the significant trait-associated SNPs. Based on similarities to existing annotated genes in other species, the putative function candidate genes that co-localized with related SNPs were annotated. The position of the highly significant SNP markers was explored by subjecting them to fine mapping and BLAST search on NCBI Genome Viewer v6.0 to annotate genomic regions and detect the nearby putative candidate genes associated with storage root formation and bulking. Putative genes within the significant SNP region were searched with respect to the significant SNP positions flanking right and left. Using the databases of the EMBL-EBI and UniProt, the functions of the genes linked to the detected SNPs were found. **Supplementary Figure S6** explains the frequency of significant SNP markers on chromosome positions. **Table 5** shows the number of SNP markers related to the number of functional genes. The result recorded most SNPs (24) and functional genes (144) for root bulking trait (RBT) and the least SNP markers (5) and functional genes (14) for FBI. **Supplementary Tables S2**–**S4** (under **Supplementary Material**) explained the putative candidate genes and their functions on the root index formation and bulking, root formation traits, and root bulking traits at different plant ages.

**Table 5:** Number of SNPs and genes significantly associated with bulking index, root formation traits, bulking traits, and ground penetrating radar (GPR) traits.

Traits	SNP	Gene
Bulking index		
Bulking index	5	14
**Total record**	**5**	**14**
Root formation		
Root color appearance	6	16
Root pedunculation	4	14
**Total record**	**10**	**30**
Bulking traits		
Dry matter content (DMC)	7	37
Dry yield	6	30
Fresh root yield (FRYD)	2	8
Harvest index (HI)	2	22
Storage root size	3	13
Starch content (SC)	4	34
**Total records**	**24**	**144**
GPR traits		
Storage root volume	2	8
Storage root biomass	3	20
Storage root diameter	1	7
**Total records**	**6**	**35**

### 3.5 Selection of the top genotypes for early root formation and bulking

**Figure 7:**
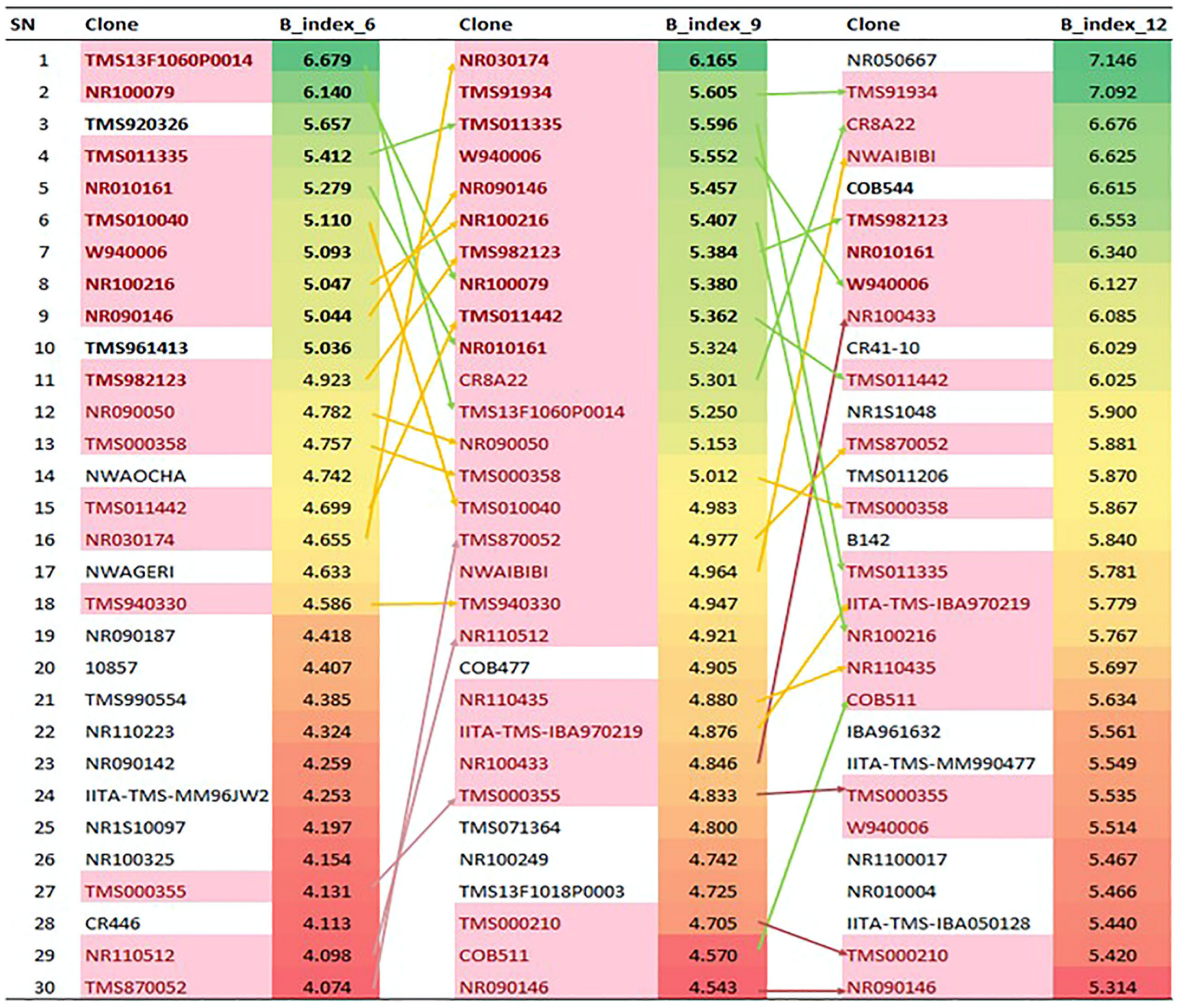
Crosstab Analysis of the relative selection index for cassava root bulking at 3, 6, 9 and 12 MAP. SN, serial number; B_index_6, _9, and _12, Formation and bulking Index at 3, 6, 9 and 12 month after planting.

The gBLUP of the bulking indices was analyzed, and a crosstab analysis of the top relative selection indices was constructed through a polynomial function of the coefficients involving the yield as the independent variable and five other bulking components (number of storage roots, root size area, storage root density, and starch and dry matter content), which had a positive significant correlation and positive direct effect on bulking of genotypes across 3, 6, 9, and 12 MAP (**Figure 7**). The means of the individual genotype across the traits were first standardized by their standard deviation after which weights were then ascribed to the independent variables based on the coefficient ranking value of the correlation analysis. The fresh root yield was given the highest weight of 100%, root size area, dry matter, and starch content (80%), while root density (70%) and the number of storage roots (60%) indicated the importance of these traits as bulking attributes. The result in **Figure 7** shows the best top 30 bulking genotypes across the harvesting age (3, 6, 9, and 12 MAP). The results showed that there were significant variations among the individual bulking index values across the harvesting age, where the genotypes behaved differently across 3, 6, 9, and 12 MAP. The 10 best-ranked genotypes for bulking value index at 6 and 9 MAP were W940006, NR090146, TMS982123, NR030174, MH944041, NR110223, COB544, TMS13F1060P0014, IITA-TMS-IBA102431, and NR010161.

The result of the principal component analysis (**Supplementary Figure S7**) also showed that genotypes W940006, NR090146, TMS982123, TMS13F1060P0014, NR010161, and COB544, represented as G220, G137, G98, G217, G196, and G111, respectively, were common and stable genotypes for the bulking index variable, which conformed to the top early root formation and bulking genotypes explained by the selection index.

The images of the cassava storage roots were viewed on **Supplementary Plate S1**. The result of images of the five selected cassava genotypes at 3, 6, 9, and 12 MAP showed promising high yield, indicating highly possible early formation and bulking at 6 and 9 MAP.

**Table 6** shows the result of the mean summary of the five best genotypes across the plant ages (3, 6, 9, and 12 MAP) for root yield and dry matter. The result showed that genotypes TMS13F1160P0004, TMS18F1279P0008, TMS18F1288P0003, TMS990558, and NR060246 were the best at 3 MAP with the range of root yield of 20.84 to 21.70 ton/ha and range of dry matter content of 32.33% to 33.78%. The five top genotypes—W940006, NR090146, TMS982123, NR030174, and MH944041—were the best at 6 MAP with root yield range of 3.01 to 7.81 ton/ha and dry matter content range of 28.49% to 30.85%. Also at 9 MAP, five genotypes—TMS18F1092P0007, COB511, NR010434, NR090146, and NR110372—were selected as the best with a root yield range of 41.73 to 73.05 ton/ha and dry matter content range of 30.00% to 33.34%. The best five genotypes at 12 MAP were NR100126, TMS011560, TMS18F1286P0003, NR1S1185, and NR110176 with root yield range of 42.80 to 63.98 ton/ha and dry matter content range of 28.95% to 38.02%.

**Table 6:** Means of the best five genotypes across the plant ages for yield and dry matter content.

Clone	Yield (ton/ha)	Dry Matter Content (%)
3 MAP		
TMS13F1160P0004	5.67	30.25
TMS18F1279P0008	3.01	29.82
TMS18F1288P0003	5.73	30.85
TMS990558	7.81	30.03
NR060246	6.42	28.49
6 MAP		
W940006	20.91	33.01
NR090146	21.67	33.56
TMS982123	21.70	32.64
NR030174	20.84	32.33
MH944041	21.23	33.78
9 MAP		
TMS18F1092P0007	73.05	31.02
COB511	49.29	33.34
NR010434	45.24	30.00
NR090146	41.73	32.39
NR110372	43.86	32.55
12 MAP		
NR100126	60.22	30.64
TMS011560	61.58	28.95
TMS18F1286P0003	42.80	38.02
NR1S1185	63.98	30.83
NR110176	60.01	32.90

## 4 Discussion

In the face of climatic changes, the choice of genotypes for ESRFB is seen as economically significant and provides a means of ensuring food security for farmers and processors. A total of 220 different cultivars from the CIAT, IITA, NRCRI, NextGen, and farmers were assessed in the current study for early storage formation of roots and bulking. To select genotypes at 6 to 9 MAP with ESRFB characteristics, a harvest basis analysis was conducted along with a calculation of the relevant heritability for each trait. This analysis looked at the dynamics of breeding values across harvest times. This study concentrated on the variables by taking into account the dynamic genetic variations and heritability of traits that can affect the early storage root bulking, formation, and final yield.

Studies on correlation enable breeders to understand the mutual relationship among traits and indirectly consider related traits that are useful for selection, which could improve genetic values (Falconer and Mackay, 1996). The coefficient of correlation of the traits showed that storage root density, root size area, number of storage roots, HI, and DRYD have a strong positive correlation with FRYD, showing interdependency (Ojulong et al., 2008), except DMC and SC, which have low correlation with fresh root yield. This suggests that any improvement in these traits will result in an increase in production, which is consistent with the findings of Ntawuruhunga et al. (2001), who found that average root weight and average number of roots per plant had a significant and favorable impact on cassava root output. According to Ntawuruhunga et al. (2001), root appearance, root form, and root pedunculation are not significant predictors of storage root yield in cassava since they are not connected with fresh root yield. An index of these significant traits (fresh root yield, root density, number of storage roots, storage root size area, and dry matter content) was derived and used as a determinant variable for storage root bulking and root formation.

The observed correlation among the bulking indices, which showed a strong positive association between 9 and 12 MAP but were not correlated with the index at 6 MAP, indicates that the bulking performance of cassava genotypes at 6 MAP varies with the performance of the genotypes at 9 and 12 MAP. The result of the correlation also means that genotypes at 6–9 MAP with high storage root density, moderately high root size area, high dry matter, high starch content, a good number of storage roots, high root volume, and good root system biomass tend to be linked to early formation and bulking. The studies by Okogbenin and Fregene (2002) and Kawano (2003) also showed that traits like harvest index and shoot mass were the most important traits associated with yield.

The analysis of variance clearly indicated significant effects of genotype and genotype by environment variations on indicator traits of early bulking and root formation. Most of the traits evaluated in this study significantly (*p*< 0.001) varied within genotypes. This suggests that the cassava genotypes evaluated had adequate genetic variability. Chipeta et al. (2016) already reported the presence of a huge diversity of agronomic traits within cassava germplasm.

Heritability estimation helps the breeder to understand if the observed variance among genotypes for the traits of interest is genetic or largely environmental. It is the proportion of the phenotype that is due to genetic control (Gowda et al., 2015). The estimates of the broad-sense heritability for the traits evaluated ranged at 0.18–0.37 for the bulking indices across plant age, 0.11–0.46 for 3 MAP, 0.10–0.42 for 6 MAP, 0.16–0.46 for 9 MAP, and 0.17–0.42 for 12 MAP, while the estimates of the broad-sense heritability for the traits ranged at 0.54–0.75 for the combined analysis, 0.17–0.77 for 6 MAP, 0.23–0.66 for 9 MAP, and 0.19–0.47 for 12 MAP. The heritability estimate was generally low to moderate and moderate to high, which was similar to the result of Yuan et al. (2019). This showed that traits with high phenotypic variance were largely genetic, which is consistent with a report by Gowda et al. (2015) and Okogbeni et al. (2013a, b). High heritability estimates are an indication that the selection of these traits should result in significant gains in cassava germplasm improvement (Clark and Watkins, 2012).

Historical complexity due to the domestication and breeding of cassava cultivars in different regions across the world may have led to diversity in the population structure of cassava over time. However, germplasm sharing across countries and regions has blunted the sharp delineation between genotypes within the global collections. Through the use of population structure analysis, genotypes could be assigned to subpopulations based on their genetic similarities assayed from a subset of SNP markers (Gapare et al., 2017). The analysis of the population structure used in this study occupied a similar genetic space of significant delta K = 2 with a differential subpopulation of 5, which was supported by a similar finding of Wolfe et al. (2016).

The number of SNP markers needed depends on the degree of LD and how LD decays with genetic distance (Myles et al., 2009) in order to obtain the highest mapping resolution possible inside the genome. The LD decay in this investigation was 1.50 kb and 0.65 kb using cut-offs of r^2^ = 0.1 and 0.2, respectively. To cover the LD between alleles in various genomic regions, GWAS, as a genetic technique, needed a large population. In spite of the advantages of GWAS for revealing genetic polymorphisms underlying agronomic traits, this approach is prone to the introduction of false positives due to population structure (Lander and Schork, 1994; Kang et al., 2008; Zhang et al., 2010). In order to avoid false-positive associations, a model based on the enhanced version of BLINK was used to exhibit significant population structure and relatedness as used by Zhou et al. (2016); Gapare et al. (2017), and Crowell et al. (2016).

This study also described the application of genome-wide association in improving ESRFB on *M. esculenta* accessions. Significant SNP markers were discovered, and putative candidate genes with their functions were also described. The use of SNP markers in classifying cassava genotypes, as well as in the discovery of putative genes in the cassava genome, has been reported by Wolfe et al. (2016). The genome-wide association (GWA) analysis was used to identify 45 significant SNP markers associated with storage root bulking and root formation. The GWAS platform was adopted because more precise physical positioning can be provided in the plant genome than the QTL mapping using just biparental mapping populations, which has been previously used for cassava bulking studies (Okogbenin and Fregene, 2002; Okogbenin et al., 2008). There were particularly high *p*-values (i.e., 7.04E−34, 7.98E−33, 1.88E−33, 1.10E−33, 2.90E−33, 1.22E−32, 5.39E−28, and 2.91E−24) recorded for bulking traits like starch content, dry matter content, and dry yield at 6 MAP, suggesting the presence of some major to moderate QTLs supporting the early bulking, while root formation traits (root color and root pedunculation) seem to be controlled by minor alleles. The rapid LD decay due to recombination caused the genome to break into smaller LD blocks so that we could fine map QTLs to the level of the gene (Olukolu et al., 2013). In this set of cassava clones, Chromosomes 2, 3, 4, 5, and 10 contained bulking trait loci and exhibited the most extensive LD. A total of 45 significant SNP markers were mapped across the four plant ages, out of which 9, 17, 7, and 12 SNPs were mapped on 3, 6, 9, and 12 MAP, respectively. The significant SNP markers were not the same for the traits across the plant ages, and most of these SNP markers were found to express more at 6 and 12 MAP.

Most of the significant SNPs associated with 6 MAP were in high LD and highly associated with bulking traits. Thus, it is possible that SNPs associated with 6 MAP are also associated with the same underlying causal variant. There were a total of five observed overlapping between the SNPs of 6, 9, and 12 MAP clusters, suggesting that SNPs identified for 6 MAP are also associated with causal SNPs controlling polymorphisms for 9 and 12 MAP. The result is in agreement with Bararyenya et al. (2020), who reported that most of the significant SNPs associated with 150 DAP of sweet potato were in high LD.

Publicly available Genome Data Viewer v6.0 (NCBI) was used to identify candidate genes encompassing or adjacent to the significant SNP markers. Several of the candidate genes that were identified play a role in the regulation of plant root growth, development, biosynthetic activities, and defense pathways. The functions of the identified genes were directly or indirectly involved in the expression of phenotypes affected by many genes with small effects. This nature of gene pattern was also reported in adaptive complex traits by Suzuki and Zool (2017). This is supported by the fact that many important agronomic traits in cassava are quantitative.

In this study, most of the functions of the putative genes discovered shared similar effects, while some of them had multiple effects (pleiotropism), which can simultaneously be favorable or unfavorable on the traits of cassava root formation and bulking. Some of the genes identified were involved in the production and regulation of growth hormones such as auxins, gibberellins, and ethylene signaling, which Bararyenya et al. (2020) have reported to regulate sweet potato lateral root development and which Kusaba et al. (2013) found to be involved in the regulation of stay-green processes in plants by maintaining greenness of leaf or by initiation and progression of leaf senescence. Ethylene is known to promote the biosynthesis of auxin, which, at low levels, promotes initiation of lateral root in the portion of the young root and, at high levels, both suppress root apical growth and inhibit lateral root initiation in root regions (Ivanchenko et al., 2008). Ethylene biosynthesis is mostly associated with the SNPs clustering with root bulking traits at 12 MAP (i.e., *Protein phosphatase 2C 53*, *Serine/threonine-protein kinase BSK7*, *DHHC-type-zinc finger*, *Cytochrome P450 78A9*, and *Receptor-like protein Cf-9 homolog*) and stress signaling pathways, whereas the cluster of genes associated with root bulking traits at 3 and 6 MAP involved mostly growth hormone signaling such as auxin, ABA, and gibberellins (i.e., *NAC domain-containing protein 100*, *Zinc finger protein 684*, *cell division cycle 23*, *NADH dehydrogenase subunit I*, *Actin-related protein 5*, and *Small polypeptide DEVIL 3*), and also genes associated with functional cellular root growth, development, and defense (i.e., *PRA1 family protein A3-like*, *Receptor-like protein 9DC3*, *endochitinase*, *E3 ubiquitin-protein ligase APD2*, *BTB/POZ and MATH domain-containing protein 3*, *Phospholipid-diacylglycerol acyltransferase 1*, *Cytosolic sulfotransferase 17-like*, *4-coumarate–CoA ligase-like 9*, *Cytokinin dehydrogenase 5*, *Calmodulin-binding transcription activator 4*, and *Histidine kinase 3*) and genes associated with biosynthesis of secondary metabolites in the plant root (i.e., *Transcription factor MYB8*, *Probable methyltransferase PMT11*, *Type I inositol polyphosphate 5-phosphatase 8*, *Cytochrome P450 78A9*, *4-coumarate-CoA ligase-like 9*, *Protein trichome birefringence-like 3*, and *NAC domain-containing protein 100*). Some of these genes have been previously identified as regulating and controlling root growth, development, and stress response in sweet potato and related crops by Bararyenya et al. (2020) and Kusaba et al. (2013). Cell proliferation and division are two complicated processes that go into storage root bulking.

The relative selection index was carried out using the crosstab BLUPs of the formation and bulking indices of the genotypes across the different plant ages. The best five genotypes selected—W940006, NR090146, TMS982123, TMS13F1060P0014, and NR010161—could help the farmers and processors to plant and harvest cassava in 6–9 months.

## 5 Conclusion

In this study, the identification of a total of 45 unique SNPs that are significantly associated with ESRFB traits with 220 cassava panels using GWAS is a significant result and tool for cassava molecular breeding. The identification of nine unique SNPs that are significantly associated with root formation and bulking traits at 3 MAP, 17 unique SNPs significantly associated with 6 MAP, seven unique SNPs significantly associated with 9 MAP, and 12 unique SNPs significantly associated with root formation and bulking traits at 12 MAP provides tools that can be used to further manipulate cassava genome at specific periods in crop development. In this study, novel genes were discovered, including 22 uncharacterized genes for ESRFB. After gene validation, these genes can be utilized to improve the genetics of cassava for ESRFB utilizing marker-assisted breeding techniques. The moderate and high heritability estimates indicate that the selection of these traits should result in significant gains in cassava germplasm improvement. The population structure analysis showed that the relatedness of the accessions will guide cassava breeders in the utilization of genetic resources for breeding purposes. The five genotypes (W940006, NR090146, TMS982123, TMS13F1060P0014, and NR010161) that were selected as the best for ESRFB could be recommended to the farmers and processors for planting and harvesting at 6 to 9 months with emphasis on W940006 as the best genotype for 6 months’ maturity.

## Data availability statement

The original contributions presented in the study are included in the article/**Supplementary Material**, further inquiries can be directed to the corresponding author/s.

## Author contributions

SA: Conceptualization, Data curation, Formal analysis, Methodology, Resources, Software, Validation, Visualization, Writing – original draft, Writing – review & editing. JOM: Methodology, Validation, Visualization, Writing – review & editing. DD: Methodology, Supervision, Visualization, Writing – review & editing. DN: Resources, Supervision, Validation, Visualization, Writing – original draft, Writing – review & editing. JO: Conceptualization, Methodology, Supervision, Validation, Visualization, Writing – review & editing. ED: Conceptualization, Investigation, Supervision, Validation, Visualization, Writing – review & editing. SO: Conceptualization, Supervision, Validation, Visualization, Writing – review & editing. PK: Conceptualization, Methodology, Supervision, Validation, Visualization, Writing – review & editing. CE: Conceptualization, Funding acquisition, Investigation, Project administration, Resources, Supervision, Validation, Visualization, Writing – original draft, Writing – review & editing.
