# Supplementary figures and images for: Determination of genomic regions associated with early storage root formation and bulking in cassava
Source: Front Plant Sci. 2024 Jun 26;15:1391452. doi: 10.3389/fpls.2024.1391452 (PMC11233741; doi:10.3389/fpls.2024.1391452)

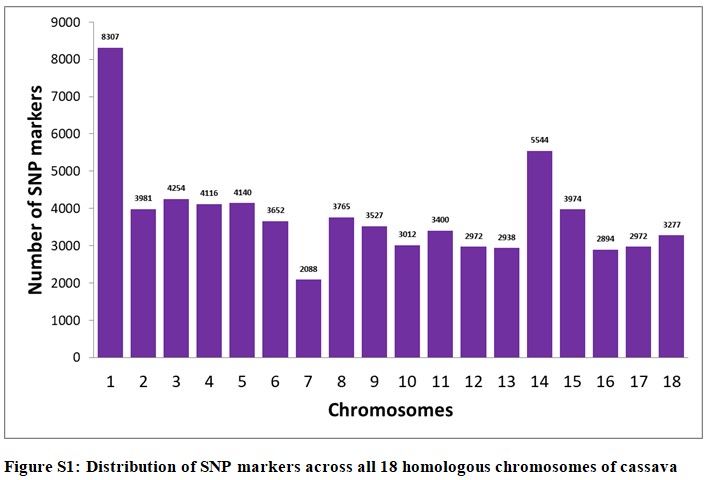

Supplement: Supplementary file 1 [file DataSheet_1.zip › Data Sheet 1/Figure S1.jpg]

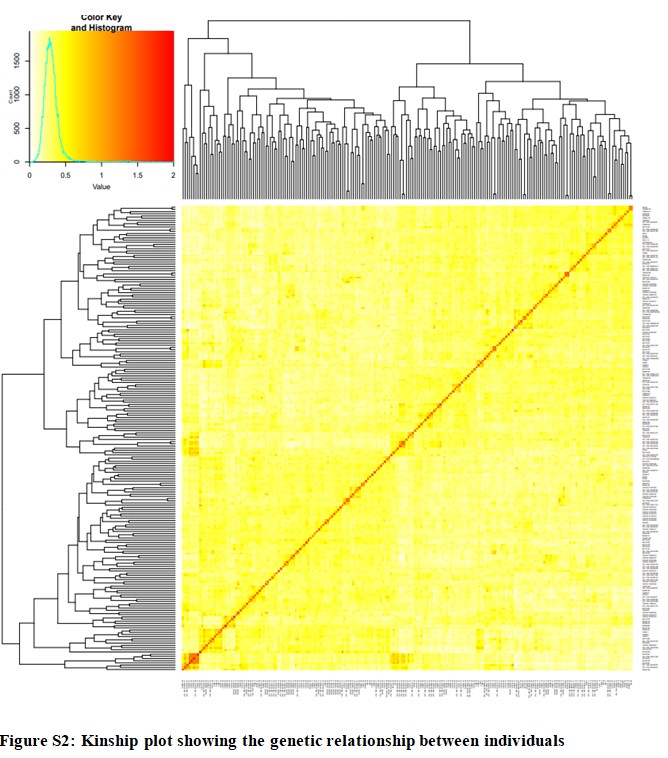

Supplement: Supplementary file 1 [file DataSheet_1.zip › Data Sheet 1/Figure S2.jpg]

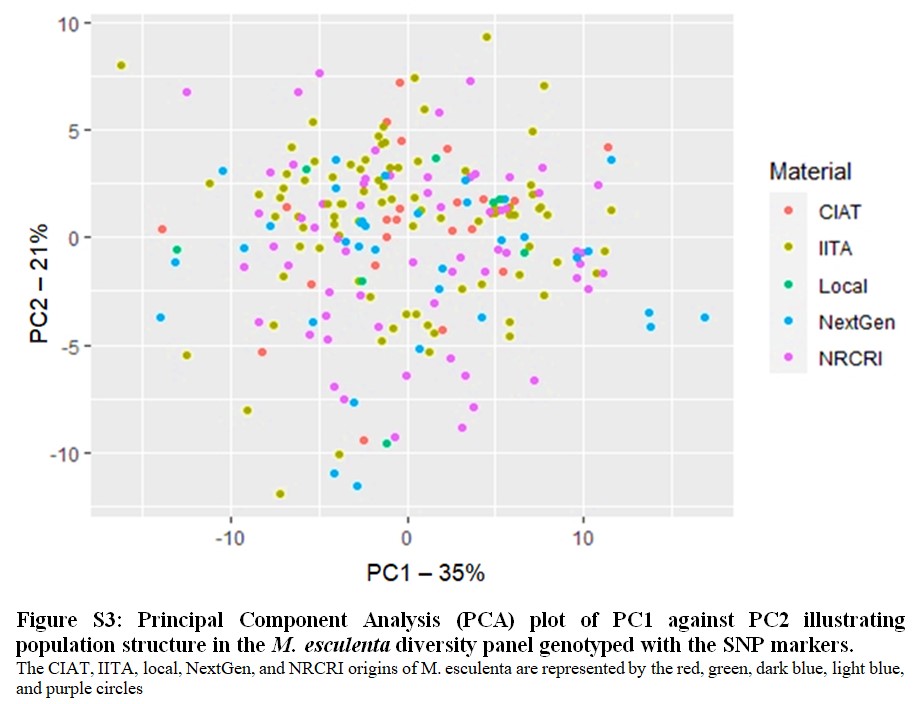

Supplement: Supplementary file 1 [file DataSheet_1.zip › Data Sheet 1/Figure S3.jpg]

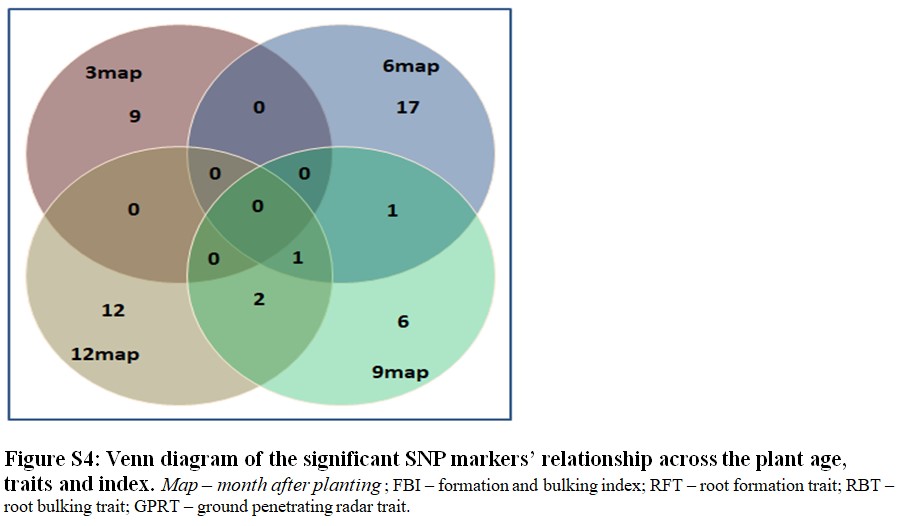

Supplement: Supplementary file 1 [file DataSheet_1.zip › Data Sheet 1/Figure S4.jpg]

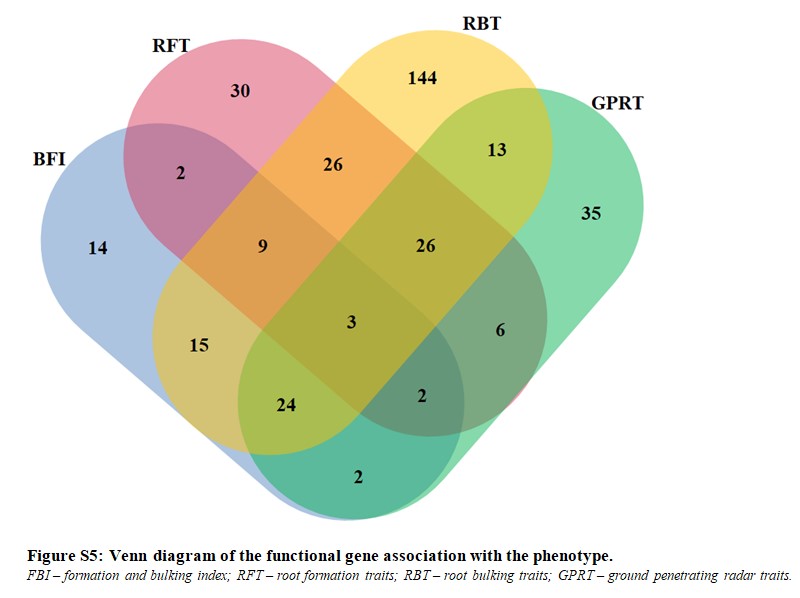

Supplement: Supplementary file 1 [file DataSheet_1.zip › Data Sheet 1/Figure S5.jpg]

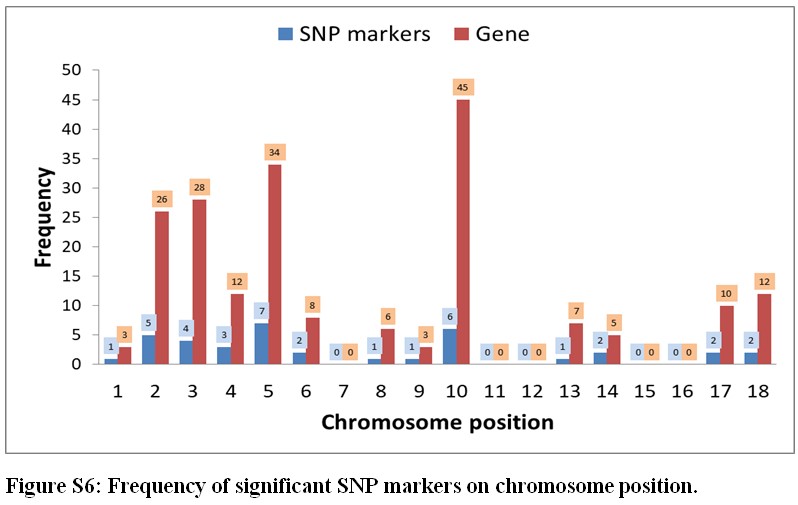

Supplement: Supplementary file 1 [file DataSheet_1.zip › Data Sheet 1/Figure S6.jpg]

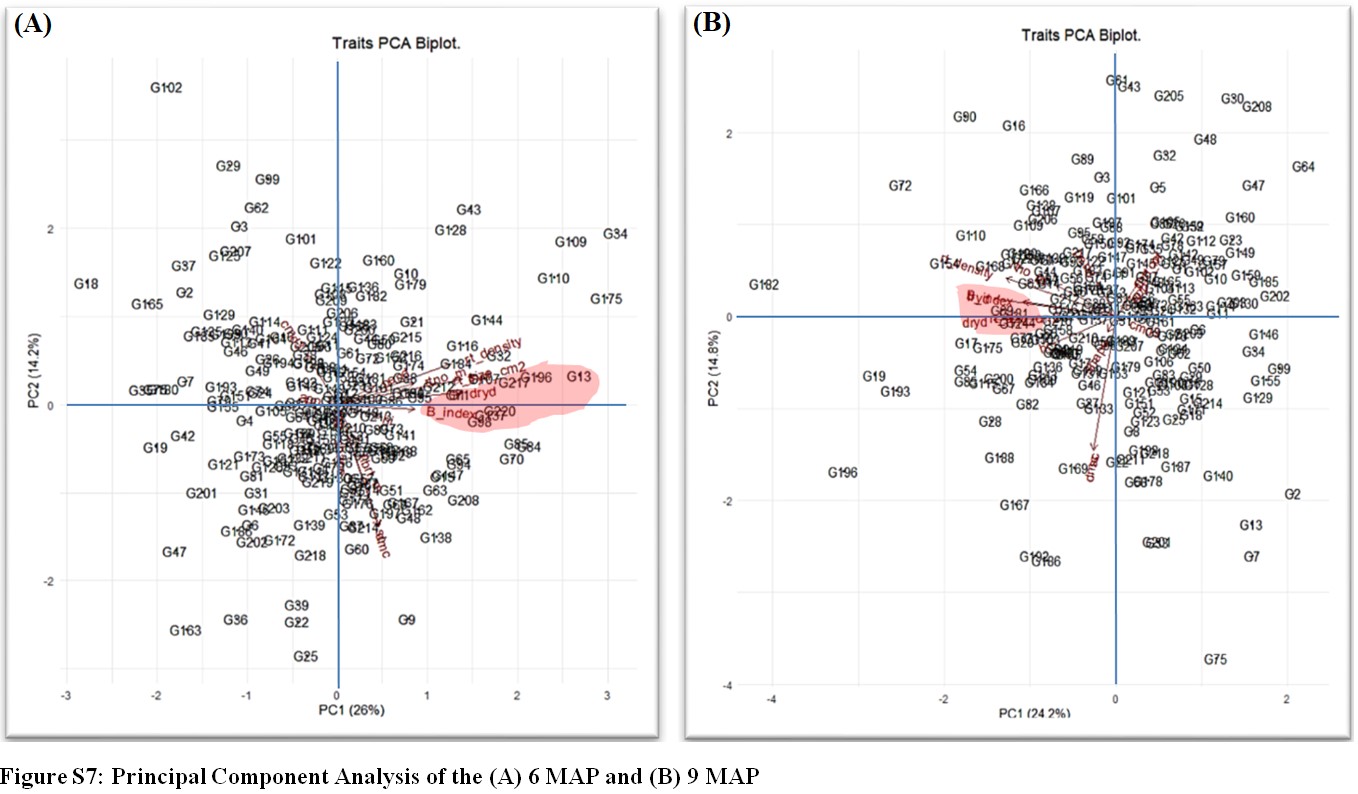

Supplement: Supplementary file 1 [file DataSheet_1.zip › Data Sheet 1/Figure S7.jpg]

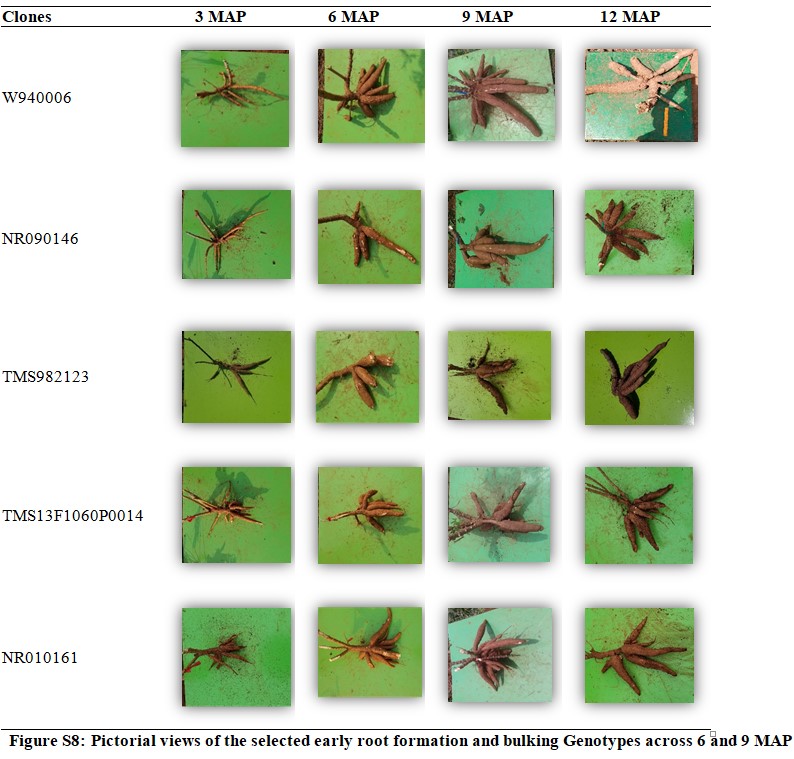

Supplement: Supplementary file 1 [file DataSheet_1.zip › Data Sheet 1/Figure S8.jpg]

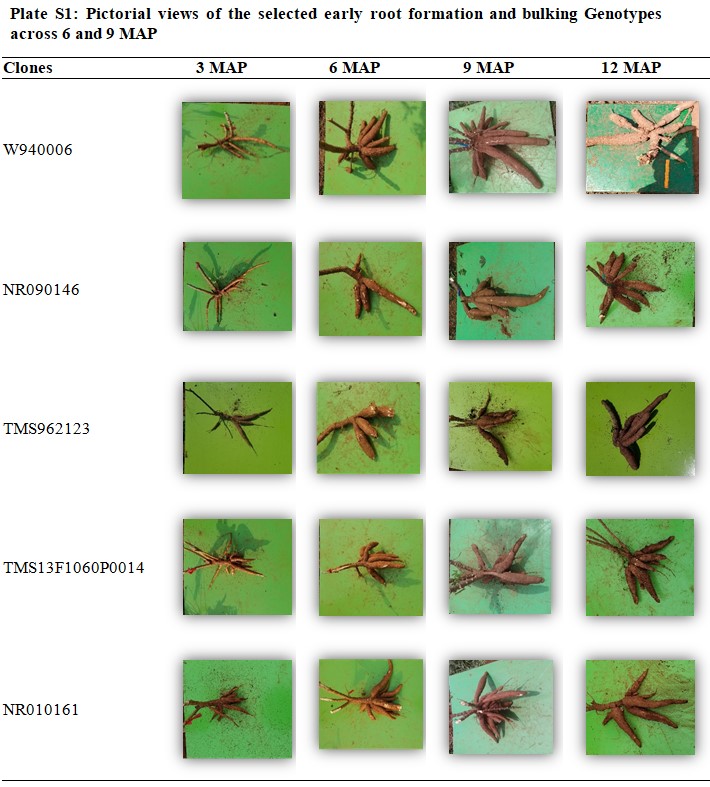

Supplement: Supplementary file 1 [file DataSheet_1.zip › Data Sheet 1/Plate S1.JPEG]
