# Supplementary material for: Determination of genomic regions associated with early storage root formation and bulking in cassava
Source: Front Plant Sci. 2024 Jun 26;15:1391452. doi: 10.3389/fpls.2024.1391452 (PMC11233741; doi:10.3389/fpls.2024.1391452)
Supplement: Supplementary file 2 [file DataSheet_2.zip › Data Sheet 2/Table S1.docx]

**Table S1: Pleiotropic SNP Markers**

| **Traits** | **Pleiotrophic SNP Marker** |
| --- | --- |
| Starch Content and Dry Matter Content | S10_2319500 |
|  | S2_1937678 |
|  | S3_3324735 |
| Dry Yield, Formation and bulking index | S4_8840623 |
