# Supplementary material for: Determination of genomic regions associated with early storage root formation and bulking in cassava
Source: Front Plant Sci. 2024 Jun 26;15:1391452. doi: 10.3389/fpls.2024.1391452 (PMC11233741; doi:10.3389/fpls.2024.1391452)
Supplement: Supplementary file 2 [file DataSheet_2.zip › Data Sheet 2/Table S2.docx]

**Table S2: Putative candidate genes and function of the root index formation and bulking at different plant age**

| **MAP** | **SNP markers** | **Chr** | **Position** | **Putative candidate gene** | **Gene description** | **Function of candidate gene** |
| --- | --- | --- | --- | --- | --- | --- |
| 3 | S5_13850266 | 5 | 13850266 | MANES_05G112755v8 LOC110607357 | NAC domain-containing protein 100 | Responsible for Ubiquitin-mediated proteolysis, auxin signaling, abiotic and biotic stress response, lateral roots, membrane anchoring, secondary wall formation, and phloem transport. |
| 3 | S5_13850266 | 5 | 13850266 | MANES_05G141152v8 LOC122723687 | Secreted RxLR effector protein 161-like | Defensive protein against effectors |
| 9 | S5_26556768 | 5 | 26556768 | MANES_05G154301v8 MANES_05G154300v8 | - NADH dehydrogenase subunit I.; - Cytochrome c oxidase subunit I; - ATP synthase F0 subunit 8; - Ubiquitin specific peptidase 4; - Peptidylprolyl isomerase H. | - Supports NADH oxidation; Catalyzes the transport of electrons from reduced cytochrome c (CYTc) to the ultimate electron acceptor, O2, in a process connected to H+ translocation for ATP synthesis. - Vital enzyme that helps the cell create ATP, which in turn releases energy for usage by the cell. - Control of the circadian clock, root meristem upkeep, jasmonate response, and plant immunology. - A cyclophilin protein controls the auxin signaling pathway and lateral root initiation. |
| 9 | S5_26556768 | 5 | 26556768 | LOC110614461 MANES_05G154600v8 | Probable ubiquitin-conjugating enzyme E2 18 | - - - Controls a wide range of cellular functions, such as transcription and epigenetic factors, enabling cells to maintain their responsiveness to signals at the cellular level and changes in the environment. |
| 9 | S1_20402446 | 1 | 20402446 | MANES _01G049500v8 | - NADH dehydrogenase subunit I.; - Cytochrome c oxidase subunit I; - ATP synthase F0 subunit 8; - Ubiquitin specific peptidase 4; - Peptidylprolyl isomerase H. | - Supports NADH oxidation; Catalyzes the transport of electrons from reduced cytochrome c (CYTc) to the ultimate electron acceptor, O2, in a process connected to H+ translocation for ATP synthesis. - Vital enzyme that helps the cell create ATP, which in turn releases energy for usage by the cell. - Control of the circadian clock, root meristem upkeep, jasmonate response, and plant immunology. - Controls the auxin signaling pathway and lateral root initiation. |
| 9 | S1_20402446 | 1 | 20402446 | MANES_01G052112v8  LOC110607807 | ORM1-like protein 1 | They have numerous functional and regulatory roles and are an essential component of plant cellular endomembranes. |
| 12 | S18_3832020 | 18 | 3832020 | MANES_18G043200v8 | Zinc finger protein 684; root cap periphery gene; cell division cycle 23 | - The growth factor for root meristems. - Homeobox in the dorsal root ganglia. - Rime used up his rooting. - Reduced lateral root development; auxin-induced root culture-like protein. |
| 12 | S18_3834291 | 18 | 3834291 | MANES_18G043200v8 LOC110606409 | Putative cyclin-A3-1 | It regulates different stages of root development and formation |
