# Supplementary material for: Determination of genomic regions associated with early storage root formation and bulking in cassava
Source: Front Plant Sci. 2024 Jun 26;15:1391452. doi: 10.3389/fpls.2024.1391452 (PMC11233741; doi:10.3389/fpls.2024.1391452)
Supplement: Supplementary file 2 [file DataSheet_2.zip › Data Sheet 2/Table S3.docx]

**Table S3: Putative candidate genes and function of the root formation traits at different plant age**

| **MAP** | **SNP** | **Chr** | **Position** | **Putative candidate gene** | **Gene description** | | **Function of candidate gene** | |
| --- | --- | --- | --- | --- | --- | --- | --- | --- |
| **Root Colour Appearance** | | | | | | | | |
| 3 | S6_21635414 | 6 | 21635414 | LOC110616577 MANES_06G081300v8 | Isoaspartyl peptidase/L-asparaginase 1 | | | Main source of nitrogen for the synthesis of proteins in tissues that are actively expanding. |
| 3 | S6_21635414 | 6 | 21635414 | LOC110617633 | hexosyltransferase GAUT11 | | | Involved in pectin and/or xylans biosynthesis in cell walls. |
| 3 | S6_21635414 | 6 | 21635414 | MANES_06G081500v8 | Zinc finger protein 684; root cap periphery gene; cell division cycle 23 | | | - The growth factor for root meristems. - Homeobox in the dorsal root ganglia. - Root culture-like protein that is activated by auxin. - Less lateral root development. |
| 6 | S3_4741499 | 3 | 4741499 | MANES_03G051100v8 | - - - Cytochrome c oxidase subunit III.     - NADH dehydrogenase subunit 4L.     - Peptidylprolyl isomerase H | | | - Represents a key metabolic function in plants. - Stages of development and growth - Controls auxin signaling and lateral root initiation |
| 6 | S3_4741499 | 3 | 4741499 | LOC110611332 LOC110611333 | Aminopeptidase M1 | | | Appears to be important in mitosis and cell division of shoot and root development. |
| 6 | S3_4741499 | 3 | 4741499 | MANES_03G050800v8 & LOC110611334 | Protein phosphatase 2C 53 | | | Important plant hormone that controls growth, development, aging and stress responses. |
| 6 | S3_4741478 | 3 | 4741478 | LOC110611332  MANES_03G051100v8 | Aminopeptidase M1 | | | Appears to be important in mitosis and cell division of shoot and root development. |
| 6 | S3_4741478 | 3 | 4741478 | MANES_03G050900v8 | - NADH dehydrogenase subunit I.; - Cytochrome c oxidase subunit I; - ATP synthase F0 subunit 8; - Ubiquitin specific peptidase 4; - Peptidylprolyl isomerase H. | | | - Supports NADH oxidation; Catalyzes the transport of electrons from reduced cytochrome c (CYTc) to the ultimate electron acceptor, O2, in a process connected to H+ translocation for ATP synthesis. - Vital enzyme that helps the cell create ATP, which in turn releases energy for usage by the cell. - Control of the circadian clock, root meristem upkeep, jasmonate response, and plant immunology. - A cyclophilin protein controls the auxin signaling pathway and lateral root initiation. |
| 9, 12 | S13_25995725 | 13 | 25995725 | LOC110607417 MANES_13G093516v8 | 50S ribosomal protein L29, chloroplastic | | | Play crucial roles in plastid ribosome function, which impacts plant development and function. |
| 9, 12 | S13_25995725 | 13 | 25995725 | LOC110607607 MANES_13G075650v8 | ubiquitin carboxyl-terminal hydrolase 4 | | | Expressed in seedlings, leaf blades, roots and stems of plant. |
| **Root Pendunculation** | | | | | | | | |
| 3 | S14_1671178 | 14 | 1671178 | LOC110630810  MANES_14G005800v8 | DDT domain-containing protein PTM | Coordinate expression of both nuclear- and chloroplast-localized genes that encode photosynthesis-related proteins. | | |
| 3 | S14_1671178 | 14 | 1671178 | MANES_14G005800v8 | - NADH dehydrogenase subunit I.; - Cytochrome c oxidase subunit I; - ATP synthase F0 subunit 8; - Ubiquitin specific peptidase 4; - Peptidylprolyl isomerase H. | - Support the oxidation of NADH; - Catalyzes the transfer of electrons from reduced cytochrome *c* (CYT*c*) to the final acceptor of electrons, O_2_, in a process that is coupled to H^+^ translocation for ATP production. - Important enzyme that provides energy to be used by the cell through the synthesis of ATP. - Regulation of plant immunity, circadian clock, root meristem maintenance and jasmonate response. - A cyclophilin protein, regulates lateral root initiation and auxin signaling pathway | | |
| 6 | S17_15171469 | 17 | 15171469 | LOC110623182 | Protein NRT1/ PTR FAMILY 1.2-like | - Acts as a nutrient and key signaling molecule coordinating gene expression, metabolism, and various growth processes throughout the plant life cycle. - Involved in transporting amino acids, nitrate, auxin (IAA), jasmonates (JAs), abscisic acid (ABA) and gibberellins (GAs) and glucosinolates | | |
| 6 | S5_15110157 | 5 | 15110157 | LOC110614930 MANES_05G132500v8 | Kinesin-like protein NACK1 | Essential for somatic cell cytokinesis, especially for the cell-plate formation and its expansion. | | |
| 6 | S5_15110157 | 5 | 15110157 | MANES_05G132600v8 | - NADH dehydrogenase subunit I.; - Cytochrome c oxidase subunit I; - ATP synthase F0 subunit 8; - Ubiquitin specific peptidase 4; - Peptidylprolyl isomerase H. - Zinc finger protein 684 - Root cap periphery gene - Cell division cycle 23 | - Support the oxidation of NADH; - Catalyzes the transfer of electrons from reduced cytochrome *c* (CYT*c*) to the final acceptor of electrons, O_2_, in a process that is coupled to H^+^ translocation for ATP production. - Important enzyme that provides energy to be used by the cell through the synthesis of ATP. - Regulation of plant immunity, circadian clock, root meristem maintenance and jasmonate response. - A cyclophilin protein, regulates lateral root initiation and auxin signaling pathway - Root meristem growth factor. - Dorsal root ganglia homeobox. - Rime spent rooting. - Auxin-induced in root culture-like protein. - Reduced lateral root formation. | | |
| 6 | S5_15110157 | 5 | 15110157 | LOC110603560 MANES_16G063400v8 | Berberine bridge enzyme-like 17 | - Contribute a significant part of the plant cell wall proteome. - Involve in the manipulation of the extracellular monolignol pool and thereby influence plant cell wall metabolism with as yet unknown implications for lignin formation | | |
| 6 | S5_15110157 | 5 | 15110157 | LOC110602919 MANES_16G063000v8 | Subtilisin-like protease SBT2.2 | - Specific functions in plant development and signaling cascades. - Functions such as development, physiology, defense and stress responses, and adaptation to the changing environment | | |
| 6 | S5_15110157 | 5 | 15110157 | MANES_16G063300v8 | - NADH dehydrogenase subunit I.; - Cytochrome c oxidase subunit I; - ATP synthase F0 subunit 8; - Ubiquitin specific peptidase 4; - Peptidylprolyl isomerase H. - Zinc finger protein 684 - Root cap periphery gene - Cell division cycle 23 | - Supports NADH oxidation; Catalyzes the transport of electrons from reduced cytochrome c (CYTc) to the ultimate electron acceptor, O2, in a process connected to H+ translocation for ATP synthesis. - Vital enzyme that helps the cell create ATP, which in turn releases energy for usage by the cell. - Control of the circadian clock, root meristem upkeep, jasmonate response, and plant immunology. - A cyclophilin protein controls the auxin signaling pathway and lateral root initiation. - The growth factor for root meristems. - Homeobox in the dorsal root ganglia. - Rime used up his rooting. - Root culture-like protein that is activated by auxin. - Less lateral root development | | |
| 6 | S5_15110157 | 5 | 15110157 | LOC110604116 MANES_16G063200v8 | Dehydrogenase/reductase SDR family member FEY | Involved in all metabolic processes related to photosynthesis as well as crucial metabolic pathways for lipid, sugar, nucleic acid, and amino acid biosynthesis or breakdown. | | |
