# Supplementary material for: Determination of genomic regions associated with early storage root formation and bulking in cassava
Source: Front Plant Sci. 2024 Jun 26;15:1391452. doi: 10.3389/fpls.2024.1391452 (PMC11233741; doi:10.3389/fpls.2024.1391452)
Supplement: Supplementary file 2 [file DataSheet_2.zip › Data Sheet 2/Table S4.docx]

**Table S4: Putative candidate genes and function of the root bulking traits at different plant age**

| **MAP** | | **SNP** | **Chr** | **Position** | **Putative candidate gene** | **Gene description** | **Function of candidate gene** |
| --- | --- | --- | --- | --- | --- | --- | --- |
| **Dry Matter Content** | | | | | | | |
| 3 | S5_1557006 | | 5 | 1557006 | LOC110615655 MANES_05G016400v8 | Conserved oligomeric Golgi complex subunit 4 | - Important for intracellular transport and glycoprotein modification; - Important for plant defense against directly penetrating fungal pathogens at the root cell. |
| 3 | S5_1557006 | | 5 | 1557006 | LOC110615708 MANES_05G016500v8 | Pyruvate dehydrogenase E1 component subunit alpha-3, chloroplastic | - Serves as the main connection between glycolysis and the tricarboxylic acid (TCA) cycle. - Plays a role in the processes that transform the energy from food into a form that cells can use. |
| 3 | S10_2912754 | | 10 | 2912754 | LOC110624725 MANES_10G029000 | Actin-related protein 5 | - Control the growth of roots. - Controls several aspects of plant morphogenesis and development, including the biological process that regulates root meristem growth. |
| 3 | S2_5069109 | | 2 | 5069109 | LOC110608741 MANES_02G065900v8 | Protein GAMETE EXPRESSED 3 | Overexpression inhibits root cell growth |
| 3 | S2_5069109 | | 2 | 5069109 | LOC110610187 MANES_02G066100v8 | Cyclin-B1-2 | - Lengthens roots more. - The growth of lateral roots and enlargement of root tips. - Influence root microtubule changes. |
| 3 | S2_10059232 | | 2 | 10059232 | LOC110609871 MANES_02G066400v8 | Transcription factor CYCLOIDEA | - Play versatile functions in multiple aspects of plant growth and development. - Help in development of diverse organs via the cell cycle |
| 3 | S2_10059232 | | 2 | 10059232 | LOC110609847 MANES_02G066500v8 | Small polypeptide DEVIL 3 | - Coordinating cellular responses necessary for differentiation, growth, and development by acting as a regulatory molecule. - Regulators of plant physiology, growth, and development. |
| 6 | S10_2319500 | | 10 | 2319500 | LOC110624967 | PRA1 family protein A3-like | - Help in role of root growth and development. - Overexpression significantly promotes root growth. - Accelerated growth and significantly increase root length. |
| 6 | S10_2319500 | | 10 | 2319500 | LOC122724935, LOC110624553, MANES_10G023900v8 MANES_10G024026v8 | Receptor-like protein 9DC3 | Increases the surface area of a root and maximizes its ability to absorb water and inorganic nutrients essential for plant growth and development. |
| 6 | S2_1937678 | | 2 | 1937678 | LOC110609737 MANES_02G021900v8 | Kinesin-like protein KIN-14J | **Transcription activation activity in regulating gibberellin biosynthesis and cell growth** |
| 6 | S2_1937678 | | 2 | 1937678 | LOC110609329 MANES_02G022100v8 | Serine/threonine-protein kinase BSK7 | - Required for plants to withstand the stress of drought. - Important plant hormone that controls cellular functions like growth, development, and abiotic stress defense responses. - Regulates the transfer of ions in guard cells. - Plants can withstand the effects of drought stress thanks to decreased transpirational water loss. |
| 6 | S3_3324735 | | 3 | 3324735 | LOC110610259 MANES_03G037700v8 | Alpha/beta hydrolase domain-containing protein WAV2 | Modulates root bending in response to environmental stimuli |
| 6 | S3_3324735 | | 3 | 3324735 | LOC110612221 MANES_03G037800v8 | Phospholipid:diacylglycerol acyltransferase 1 | Play an overlapping roles in triacylglycerol (TAG) assembly  Contributes to TAG accumulation. |
| 6 | S3_3324735 | | 3 | 3324735 | LOC110612414 MANES_03G037600v8 | protein PLASTID MOVEMENT IMPAIRED 1-RELATED 1 | Play important roles in fundamental cellular activities and adaptive responses to environmental stress in plants. |
| 6 | S3_3324735 | | 3 | 3324735 | LOC110612104 MANES_03G037900v8 | Protein Brevis radix-like 4 | Identified as a modulator of root growth. |
| 6 | S3_3324735 | | 3 | 3324735 | LOC110611194 MANES_03G038000v8 | protein BIG GRAIN 1-like A | - Plays a role in grain development, plant growth, and gravitropism. - Increase plant yield, seed weight, and biomass. - Expands the size of the main organs and roots. - A crucial developmental protein used in the construction of the roots. - Take part in stress tolerance and yield component regulation |
| 6 | S3_3324735 | | 3 | 3324735 | LOC110612102 MANES_03G038200v8 | RING-H2 finger protein ATL1 | Play significant roles in plant growth, development, stress resistance, and signal transduction. |
| 6 | S3_3324735 | | 3 | 3324735 | LOC110610463 MANES_03G038300v8 | Mechanosensitive ion channel protein 10 | Diverse roles in the formation of pollen tubes, control of plastid shape, seed germination, and root development in plants. |

| **Dry Yield** | | | | | | | | |
| --- | --- | --- | --- | --- | --- | --- | --- | --- |
| 6 | S6_19749539 | | 6 | | 19749539 | LOC110618340 MANES_06G063000v8 | protein DETOXIFICATION 14 | Involved in nitrogen detoxification Impact on Protein Synthesis and Root Development in Cassava Roots. |
| 6 | S6_19749539 | | 6 | | 19749539 | LOC110617342 MANES_06G062900v8 | ATP synthase subunit beta, mitochondrial | - Synthesizes the main bulk of cytosolic ATP. - Converts the electrochemical proton gradient into ATP. |
| 6 | S17_18894518 | | 17 | | 18894518 | LOC110604801 MANES_17G023800v8 | Probable prolyl 4-hydroxylase 6 | - Catalyze an important post-translational modification in plants**.** - Required for proper cell wall self-assembly and hence root hair elongation |
| 6 | S17_18894518 | | 17 | | 18894518 | LOC110605470 MANES_17G023900v8 | Transcription factor MYB35 | - Controls root epidermal cell specification. - Inhibit root hair (RH) formation by activating the GL2 gene expression. |
| 6 | S17_13553658 | | 17 | | 13553658 | LOC110605409 MANES_17G022300v8, LOC110605499, MANES_17G022600v8 | V-type proton ATPase subunit d2. | - Required for efficient nutrient storage. - Plays an important role in plant growth |
| 6 | S17_13553658 | | 17 | | 13553658 | LOC110605174 MANES_17G022200v8 | Probable E3 ubiquitin ligase complex SCF subunit sconB | A variety of biological processes, including hormonal Control of biotic and abiotic stress tolerance, light response, plant reproduction, vegetative development, and DNA repair |
| 6 | S4_26245979 | | 4 | | 26245979 | LOC110613858 MANES_04G065900v8 | Endochitinase | Play a part in the processes of defense, growth, and development.  Boost plant output and growth. |
| 6 | S4_26245979 | | 4 | | 26245979 | LOC110613855 MANES_04G066000v8 | Probable inactive chitinase-like protein LaCIC | Play multiple roles in defense, development and growth regulation in plants. |
| 12 | S4_8840623 | | 4 | | 8840623 | LOC110612547  MANES_04G050700v8 | Diazcylglycerol lipase-beta | - Enhance membrane stability. - Function in stress tolerance and the provision of substrates for the biosynthesis of bioactive compounds. |
| 12 | S4_8840623 | | 4 | | 8840623 | LOC110613962 MANES_04G054500v8 | Transcription factor bHLH128 | - Control the plant's adaptive reactions - Impact on the development and production of important crops. - In closed chromatin, crucial elements for cellular reprograming are often found. |
| 12 | S18_3834291 | | 18 | | 3834291 | LOC110607026 MANES_18G043300v8 | Agamous-like MADS-box protein AGL66 | - Control of lateral organ development and flowering time. - Regulates root meristem cell division and promotes overall root vascular tissue formation |
| 12 | S18_3834291 | | 18 | | 3834291 | LOC110606009 MANES_18G043400v8 | E3 ubiquitin-protein ligase APD2 | - Importance in controlling cellular functions. - Control plant development and growth - Developed a wide range of intricate adaptive mechanisms to deal with unfavorable environmental circumstances, including as the preservation of ion homeostasis, accumulating antioxidant enzymes, and producing suitable products. |
| 12 | S18_3834291 | | 18 | | 3834291 | LOC110606409 MANES_18G043200v8 | Putative cyclin-A3-1 | Cell cycle regulation, cell death control, and DNA repair |
| 12 | S18_3834291 | | 18 | | 3834291 | LOC110606712 MANES_18G043000v8 | Cytochrome P450 81C13 | - It aids in carotenoid pigment formation, which is important for photosynthesis and photoprotection. - Assuring mechanical support, water retention, and transport by serving as the building blocks for structural biopolymers such lignin, suberin, cutin, and sporopollenin. - Participate in the growth, development, or fitness of plants. |
| **Fresh Root Yield** | | | | | | | | |
| 6 | | S18_9930952 | | 18 | 9930952 | LOC110605839 MANES_18G103400v8 | DEAD-box ATP-dependent RNA helicase FANCM. | - Involved in ordered homologous recombination (HR) events in somatic and meiotic cells. - Involved in the suppression of spontaneous HR events in somatic cells. - Associate with a diverse range of cellular functions including response to abiotic stress. |
| 6 | | S18_9930952 | | 18 | 9930952 | LOC110606107 MANES_18G103600v8 | Transcription factor MYB8 | - Involved in the regulation of secondary wall biosynthesis. - Activate the biosynthetic pathways of cellulose, xylan and lignin. |
| 12 | | S4_8840623 | | 4 | 8840623 | LOC110614192 MANES_04G053400v8 | Galactolipase DONGLE, chloroplastic | Play pivotal roles during plant developmental processes, such as seed maturation, viable pollen production, root growth, and tendril coiling, and they also function as important signaling molecules in plant defense responses to biotic and abiotic stress. |
| 12 | | S4_8840623 | | 4 | 8840623 | LOC110612547 MANES_04G050700v8 | Diacylglycerol lipase-beta | - Enhance membrane stability. - Function in stress tolerance and the provision of substrates for the biosynthesis of bioactive compounds. |
| **Harvest Index** | | | | | | | | |
| 3 | | S14_4092696 | | 14 | 4092696 | LOC110600339 MANES_14G047200v8 | Proteasome subunit alpha type-4 | Increased cell sizes, decreased heat shock tolerance, increased oxidative stress tolerance. |
| 12 | | S10_2601853 | | 10 | 2601853 | LOC110630745 MANES_14G047100v8 | Ubiquitin-conjugating enzyme E2 27 | Important role in plant development, growth, and external stress responses |
| 12 | | S10_2601853 | | 10 | 2601853 | LOC110600271 MANES_14G046800v8 | CASP-like protein 1F1. | - Genes involved in plant defense as well as growth promotion and regulation of specific processes (flowering, photosynthesis, glucose catabolism, and root growth). - Increasing plant weight, crop yield, and seed germination |
| 12 | | S10_2601853 | | 10 | 2601853 | LOC110624115 MANES_10G026300v8 | Protein HESO1 | Help in root architecture, root length and root hairs development. |
| 12 | | S10_2601853 | | 10 | 2601853 | LOC110624114 MANES_10G026200v8 | Probable methyltransferase PMT11 | Several secondary metabolites, such as phenylpropanoids, flavonoids, and alkaloids, methylate the oxygen atom. |
| 12 | | S10_2601853 | | 10 | 2601853 | LOC110624586, MANES_10G026500v8, LOC110625324 MANES_10G026600v8 | Tropinone reductase homolog At5g06060 | Predominantly trigger signals for plant development |
| 12 | | S10_2601853 | | 10 | 2601853 | LOC110625187 MANES_10G026400v8 | Protein phosphatase 2C 37 | Function as regulators of various signal transduction pathways. |
| 12 | | S10_2601853 | | 10 | 2601853 | LOC110624973 MANES_10G025950v8 | BTB/POZ and MATH domain-containing protein 3 | Involved in plant growth and development. |
| 12 | | S10_2601853 | | 10 | 2601853 | LOC110625323 MANES_10G026700v8 | Receptor-like protein Cf-9 homolog | Play key roles in **plant** defense and development |
| 12 | | S10_2601853 | | 10 | 2601853 | LOC110624466 MANES_10G024900v8 | CASP-like protein 5B2 | - In root systems is involved in stress resistance and maintaining homeostasis. - Regulatory role on lateral root growth and development |
| **Storage root size** | | | | | | | | |
| 9 | | S9_26051761 | | 9 | 26051761 | LOC110608208 MANES_09G090321v8 | Type I inositol polyphosphate 5-phosphatase 8. | - Functions in various aspects of plant growth and development. - Required for secondary wall synthesis and actin organization in fiber cells |
| 9 | | S9_26051761 | | 9 | 26051761 | LOC122724719 | Small nucleolar RNA R71. | Manipulate plant metabolites, develop plants with improved resistance to environment stresses, and engineer plants to defend against pathogen infections. |
| 9 | | S5_12439769 | | 5 | 12439769 | LOC110615234 MANES_05G118900V8 | Pentatricopeptide repeat-containing protein At4g20740 | Involved in RNA regulation and metabolism in plant organelles. |
|  |  |  |  |  |  | LOC110615220 MANES_05G118701v8 | Uncharacterized protein |  |
| 9 | | S2_4134534 | | 2 | 4134534 | LOC110609508 MANES_02G051800v8 | Probable galacturonosyltransferase 13 | - Function redundantly in pollen tube growth, possibly via taking part in pectin biosynthesis of the pollen tube wall. - Catalyzes the elongation of HG oligogalacturonides in a -1, 4-configuration.. |
| 9 | | S2_4134534 | | 2 | 4134534 | LOC110608705 MANES_02G051600v8 | Chaperone protein ClpB3, chloroplastic | - Important for the growth of chloroplasts and seedling survival - Plays a role as a molecular chaperone in plastid differentiation, mediating the creation of internal thylakoid membrane and providing chloroplasts with thermotolerance under heat stress.. |
| 9 | | S2_4134534 | | 2 | 4134534 | LOC110603286 MANES_02G052200v8 | Alpha-1,4 glucan phosphorylase L isozyme, chloroplastic/amyloplastic | Largely known for the phosphorolytic degradation of starch. |
| **Starch Content** | | | | | | | | |
| 3 | | S5_1735523 | | 5 | 1735523 | LOC110614889 MANES_05G018900v8 | Cytochrome P450 78A9 | - Functions as diverse catalysts and is essential for the production of secondary metabolites, antioxidants, and phytohormones in higher plants. - Plays a key role in the detoxification of xenobiotics. |
| 3 | | S5_1735523 | | 5 | 1735523 | LOC110614546, MANES_05G018950v8, LOC110615807 MANES_05G018800v8 | Uncharacterized membrane protein At1g16860 | - Take part in a variety of physiological processes in plants, such as energy conversion and material transport. - assist in the transfer of various substrates, metabolites, signaling molecules, and phytohormones between cells. - Ion channels in transmembrane proteins move or eliminate ions and harmful chemicals from cells. - serve as enzymes by detecting chemical signals in the surroundings and transmitting them to the interior of the cell |
| 3 | | S5_1735523 | | 5 | 1735523 | LOC110615808 MANES_05G018700v8 | Homeobox-leucine zipper protein ATHB-12 | Regulates leaf growth by promoting cell expansion and endoreduplication |
| 6 | | S10_2319500 | | 10 | 2319500 | LOC110624970, MANES_10G024000v8, LOC110625096 MANES_10G024240v8 | PRA1 family protein A3-like | Small transmembrane proteins that operate as VAMP2 and the vacuolar soluble N-ethylmaleimide-sensitive factor attachment receptor protein Rab GTPase receptors to control vesicle trafficking. |
| 6 | | S10_2319500 | | 10 | 2319500 | LOC110625098 MANES_10G024300v8 | Receptor-like protein Cf-9 homolog | Primarily as receptor-like proteins or receptor-like kinases, conferring recognition of numerous pathogen compounds and plant hormones, play important roles in plant defense and development. |
| 6 | | S10_2319500 | | 10 | 2319500 | LOC110624870 MANES_10G023400v8 | Folylpolyglutamate synthase | By catalyzing the conversion of folates to polyglutamate derivatives, it enables intracellular retention of these cofactors, which are essential substrates for the majority of folate-dependent enzymes involved in one-carbon transfer processes involved in purine, pyrimidine, and amino acid synthesis. |
| 6 | | S2_1937678 | | 2 | 1937678 | LOC110609737 MANES_02G021900v8 | Kinesin-like protein KIN-14J | - In charge of conveying a variety of cargos unidirectionally, such as mRNAs, protein complexes, and membrane organelles. - Play important functions in signal transduction, morphogenesis, and mitosis. |
| 6 | | S2_1937678 | | 2 | 1937678 | LOC110609329 MANES_02G022100v8 | Serine/threonine-protein kinase BSK7 | Acts as a "central processor unit" (cpu), taking input data from receptors that detect environmental stimuli, phytohormones, and other external events, and converting that data into appropriate outputs, such as changes in metabolism, gene expression, and cell growth and division.. |
| 6 | | S2_1937678 | | 2 | 1937678 | LOC110610053 MANES_02G021800v8 | Zinc finger protein CONSTANS-LIKE 2 | - Associated with plant architecture, abiotic stress response, and auxin homeostasis - Involved in flowering time regulation |
| 6 | | S2_1937678 | | 2 | 1937678 | LOC110609607 MANES_02G022200v8 | Glutamate--cysteine ligase, chloroplastic | Help in the normal plant development and stress tolerance. |
| 6 | | S2_1937678 | | 2 | 1937678 | LOC110609453 MANES_02G021700 | General transcription and DNA repair factor IIH helicase subunit XPB1 | - Initiation transcription of protein- coding gene. - DNA nucleotide repairing. |
| 6 | | S3_3324735 | | 3 | 3324735 | LOC110610259, MANES_03G037700v8 | Alpha/beta hydrolase domain-containing protein WAV2 | These enzymes function as esterases, thioesterases, lipases, proteases, dehalogenases, and epoxide hydrolases, catalyzing both primary and specialized (secondary) metabolism.. |
| 6 | | S3_3324735 | | 3 | 3324735 | LOC110612414 MANES_03G037600v8 | Protein PLASTID MOVEMENT IMPAIRED 1-RELATED 1 | Plays a crucial part in mesophyll cell movement caused by chloroplast photorelocation mediated by cp-actin. |
| 6 | | S3_3324735 | | 3 | 3324735 | LOC110612221 MANES_03G037800v8 | Phospholipid:diacylglycerol acyltransferase 1. | - Good for oil yield increase. - Helps in the growth and development of root, leaf, stem, flower, and seeds. |
